# An Improved Unscented Kalman Filter Based Decoder for Cortical Brain-Machine Interfaces

**DOI:** 10.3389/fnins.2016.00587

**Published:** 2016-12-22

**Authors:** Simin Li, Jie Li, Zheng Li

**Affiliations:** ^1^State Key Laboratory of Cognitive Neuroscience and Learning and IDG/McGovern Institute for Brain Research, Beijing Normal UniversityBeijing, China; ^2^Center for Collaboration and Innovation in Brain and Learning Sciences, Beijing Normal UniversityBeijing, China

**Keywords:** brain-machine interface, neural decoding, encoding model, unscented Kalman filter, brain-computer interface, neuroprosthetic

## Abstract

Brain-machine interfaces (BMIs) seek to connect brains with machines or computers directly, for application in areas such as prosthesis control. For this application, the accuracy of the decoding of movement intentions is crucial. We aim to improve accuracy by designing a better encoding model of primary motor cortical activity during hand movements and combining this with decoder engineering refinements, resulting in a new unscented Kalman filter based decoder, UKF2, which improves upon our previous unscented Kalman filter decoder, UKF1. The new encoding model includes novel acceleration magnitude, position-velocity interaction, and target-cursor-distance features (the decoder does not require target position as input, it is decoded). We add a novel probabilistic velocity threshold to better determine the user's intent to move. We combine these improvements with several other refinements suggested by others in the field. Data from two Rhesus monkeys indicate that the UKF2 generates offline reconstructions of hand movements (mean CC 0.851) significantly more accurately than the UKF1 (0.833) and the popular position-velocity Kalman filter (0.812). The encoding model of the UKF2 could predict the instantaneous firing rate of neurons (mean CC 0.210), given kinematic variables and past spiking, better than the encoding models of these two decoders (UKF1: 0.138, p-v Kalman: 0.098). In closed-loop experiments where each monkey controlled a computer cursor with each decoder in turn, the UKF2 facilitated faster task completion (mean 1.56 s vs. 2.05 s) and higher Fitts's Law bit rate (mean 0.738 bit/s vs. 0.584 bit/s) than the UKF1. These results suggest that the modeling and decoder engineering refinements of the UKF2 improve decoding performance. We believe they can be used to enhance other decoders as well.

## Introduction

Brain-machine interfaces (BMIs) have the potential to improve the well-being of people with paralysis, locked-in syndrome, and other ailments, as well as change how humans interact with machines and each other. While there has been substantial progress (Baranauskas, 2014; Nuyujukian et al., 2015) in the accuracy or communication bandwidth of BMI, there is still room for improvement. In our previous work with the unscented Kalman filter based decoder (Li et al., 2009), which we refer to as UKF1, we proposed a non-linear model of neural tuning which modeled the relationship between spike counts and the position and velocity of a cursor. Since that study, much progress has been made in decoder engineering and motor cortical encoding models. We have collected several novel modeling and decoder engineering refinements, as well as incorporated work from others, to form an improved unscented Kalman filter based decoder, which we call UKF2.

The refinements to the encoding model can be summarized as adding neural tuning to hand acceleration, hand position and velocity in an interactive term, and target position and modeling neuron autocorrelation and cross-neuron correlation using spiking history. We include target position as a decoded variable, i.e., the UKF2 does not require knowledge of the true target position to operate. The refinements to decoder engineering are the use of a combination of position and velocity estimates to control the cursor, probabilistically thresholding velocity to determine when the user wishes to remain still, and using estimates of future intended movement to drive the cursor. We do not modify the unscented Kalman filter algorithm itself; rather, our improvements are in the design of the filter's observation model and post-processing of filter outputs.

Using data from two Rhesus monkeys, we compare UKF2 to UKF1, as well as the popular position-velocity Kalman filter in terms of offline reconstructions of hand-controlled cursor movement, encoding model predictive power, and closed-loop neural control of cursor. We also examine the contributions of each modeling refinement. Our results show that the UKF2 reconstructs hand-controlled cursor movement more accurately than the position-velocity Kalman filter and the UKF1. Our analysis suggest that the encoding model of the UKF2 encodes neural activity better, as evidenced by better predictions of firing rate given kinematic and past spiking information. Our analysis of the modeling refinements indicates that spiking history contributed the most to encoding accuracy, but hand acceleration and target position contributed most to decoding accuracy. Finally, experiments in which monkeys used the decoders in closed-loop neural control of the cursor showed that, using the UKF2, monkeys could complete a center-out task significantly faster and with higher Fitts's Law bit rate than using the UKF1, and UKF2 performance was comparable to the FIT Kalman filter (Fan et al., 2014).

Our results indicate that the enhancements of the UKF2 improved the functionality of the decoder. Some of the enhancements, such as modeling of hand acceleration, target position, as well as the probabilistic thresholding of velocity, can be readily used by the Kalman filter and similar decoding algorithms.

## Materials and methods

### Surgical procedures

**Figure 1:**
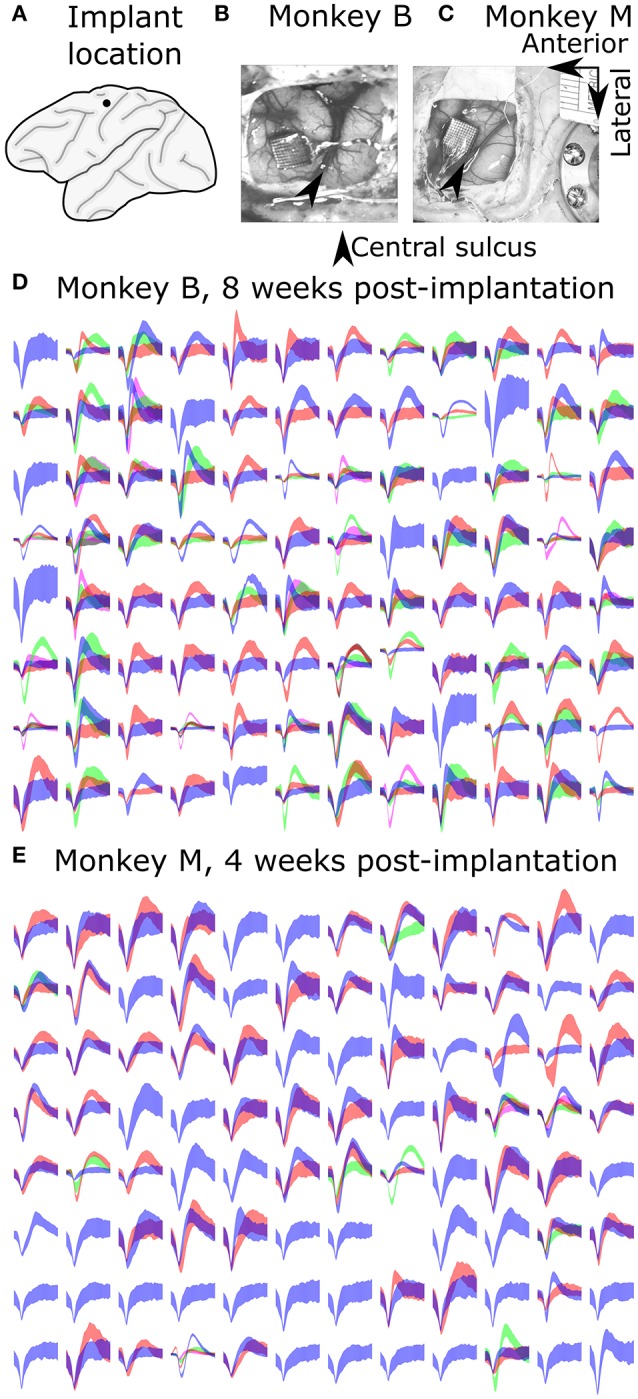
**Array implant locations and example recorded signals. (A)** Implant location. **(B,C)** Photos taken during surgeries. **(D,E)** Sample waveforms. Each sub-panel shows waveforms from one channel, drawn as mean ± one standard deviation.

All surgical procedures were in compliance with the U.S. National Institutes of Health Guide for the Care and Use of Laboratory Animals and were approved by the Institutional Animal Care and Use Committee of Beijing Normal University. Two adult male (6 years, 11 kg; 4 years, 8 kg) Rhesus monkeys (*Macaca mulatta*) were implanted with silicon-based electrode arrays (Utah array, Blackrock Microsystems) in the left primary motor cortex under sterile conditions. We followed standard Utah array implantation procedures. In each animal, a Utah array was implanted approximately 4 mm anterior to the central sulcus, at approximately 15 mm lateral from the midline (Figure 1A), targeting arm and hand areas. Photos from the implantation surgeries for monkey B and monkey M are shown in Figures 1B,C, respectively.

### Electrode array

We used 96-channel Utah arrays (Blackrock Microsystems) with 1.0 mm long electrode shanks (monkey B) or 1.5 mm shanks (monkey M). The arrays were arranged in a 10 × 10 grid pattern with inter-electrode separation of 400 um. The shank material was silicon with platinum coating on the electrode tip and polyimide insulation (Jones et al., 1992). Electrode diameter tapered from 80 um to a fine point (Jones et al., 1992).

### Signal acquisition and processing

Signals were recorded from the Utah array using a Plexon Omniplex recording system in an experiment room shielded from electromagnetic interference. Signals were amplified (up to 8000x), digitized (at 16 bit, 40 kHz), and processed in the Omniplex system. A desktop personal computer (Dell Precision T3500 with an Intel Xeon W3565 3.2 GHz processor and 8 GB RAM) received the processed signals and executed the Plexon PlexControl software, as well as our experimental control and decoding software.

Spikes were detected and sorted in real time using the Omniplex hardware. The spike detection threshold and sorting parameters were set by visual inspection by the experimenter using Plexon's software. Both well-isolated single units and multiunits were used for decoding and analysis, and subsequently referred to as units without distinction.

We aggressively spike sorted, that is, we preferred to differentiate waveforms into a larger number of units when the choice was not obvious. We often sorted several multiunits per channel. Our reasoning was that if we mistakenly split the waveforms from one neuron into two units, the model fitting should not be biased (though noise due to variance would increase during decoding). If we put two neurons in the same unit, barring a specially designed decoding algorithm such as the switching Kalman filter (Wu et al., 2004), we would lose information. We illustrate the sorted spike waveforms from all channels of monkey B and monkey M in Figures 1D,E, respectively.

After spike sorting, spikes were counted in 50 ms duration, non-overlapping bins to estimate the instantaneous firing rate of each unit. All decoders and parameter fitting used this spike count for input.

### Experiment control and kinematics measurement

A custom brain-machine interface software suite (BMI3, Nicolelis Lab, Duke University) performed experiment control, model fitting, and real time decoding. This software suite communicated with the Plexon software via Plexon's C language application program interface.

**Figure 2:**
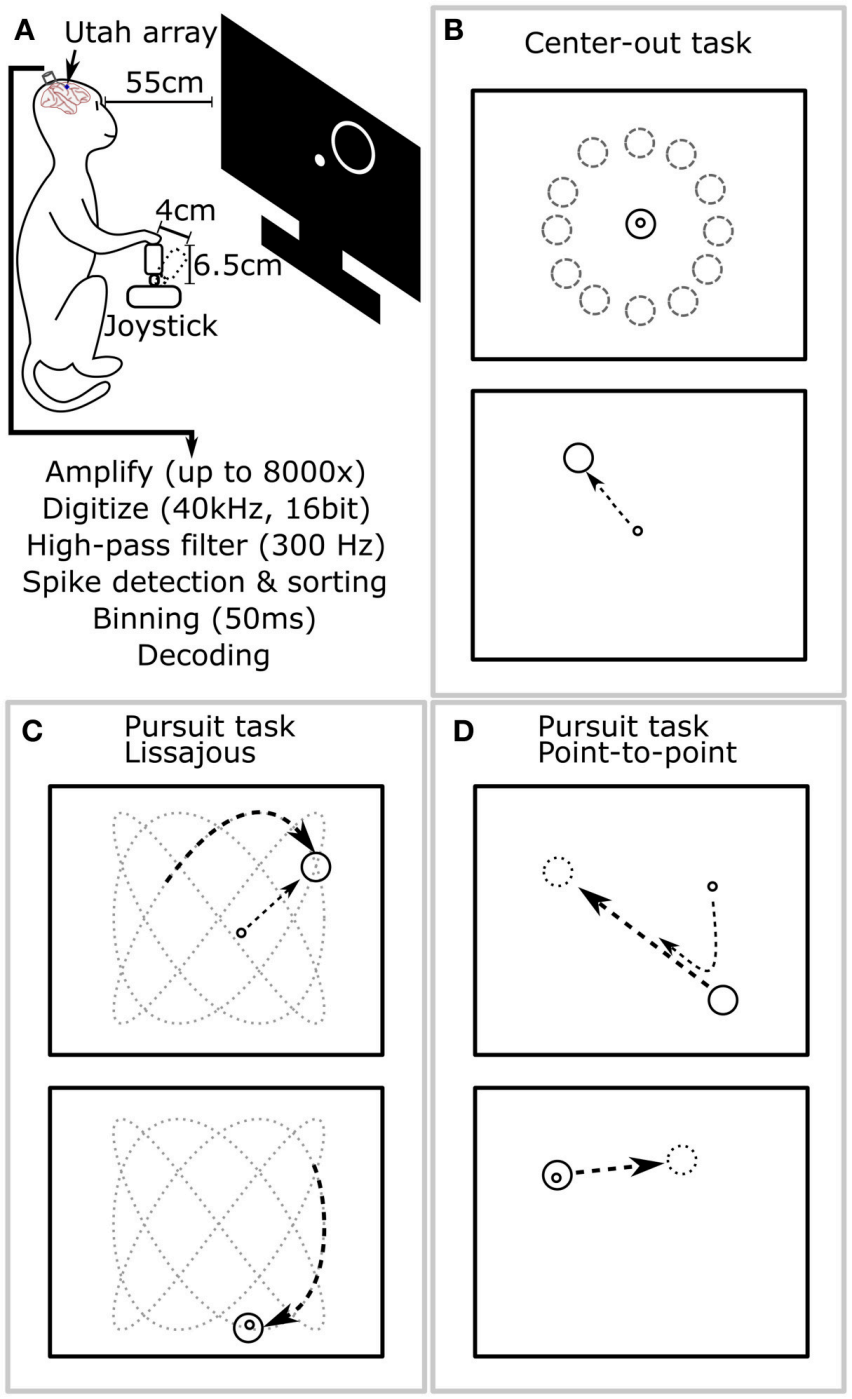
**Experimental setup and behavioral tasks. (A)** The monkey sat in a primate chair 55 cm before a computer screen and grasped a 6.5 cm tall joystick with 4 cm maximum deflection in its right hand. **(B)** Center-out task. The monkey alternatively moved the cursor to center targets and peripheral targets, located at random angles and fixed distance from center. **(C)** Pursuit task with Lissajous curve: the monkey kept the cursor within a target which moved continuously following a Lissajous curve. **(D)** Pursuit task with point-to-point trajectory: the monkey kept the cursor within a target which moved continuously between randomly selected points on the screen.

An analog 3-axis potentiometer joystick (CH-400R-P3, Hangzhou Chuang Hong Electric Co.) was used to capture hand motion data. Only the x and y axes were used, and the rotation axis was ignored in these experiments. The length of the joystick, including handle, was 6.5 cm, and the maximum deflection of the joystick was approximately 4 cm (Figure 2A). The joystick was self-centered by a weak spring. This joystick was smaller than that used in our previous work (Li et al., 2009); we observed that the monkey primarily moved its elbow and shoulder joints to control the joystick.

The joystick was connected to a PCI-DAS1002 analog-to-digital recording card (Measurement Computing) mounted in a separate desktop personal computer (Dell Precision T3500). Custom software read the joystick measurements and sent them to BMI3 using a gigabit Ethernet local area network.

The position of the joystick was mapped to the position of the cursor on the screen in a one-to-one, piecewise-linear manner, with forward (anterior) joystick positions mapped to upper screen locations and backward (posterior) joystick positions mapped to lower screen locations. Joystick movements and on-screen cursor movements had a scaling ratio of approximately 1:4. Joystick measurements were recorded at 100 Hz. To match the 50 ms bin size of spike counts, the average of the five joystick position measurements within each bin was used.

### Behavioral tasks

During experiments, the monkey sat in a primate chair and a flat panel computer monitor was placed 55 cm in front of it (Figure 2A). Prior to experiments, all monkeys were trained on two behavioral tasks: center-out and pursuit. In the center-out task (Figure 2B), monkeys had to move a circular cursor (logically 0 cm diameter) into a circular target (5 cm diameter) which alternatively appeared in the screen center and the periphery. The peripheral locations were equidistant (8–10 cm) from the screen center at a random angle. Hold time was set to 500 ms. In the pursuit task, monkeys had to keep the cursor within a continuously moving target (6 cm diameter). The target moved according to a Lissajous curve (Figure 2C) or a smoothed point-to-point trajectory (Figure 2D). The center-out task was used for offline and closed-loop decoding. The pursuit task was only used for offline decoding. Details of the tasks can be found in the Supplementary Materials.

### Algorithmic overview

Our decoding method is based on the n-th order unscented Kalman filter decoder (Li et al., 2009), but with numerous enhancements, some novel and some based on prior work. The enhancements fall into two broad categories: those that modify the neural encoding model and those that modify the control mechanism during closed-loop neural control. Encoding model refinements help both offline reconstructions and closed-loop neural control, while control mechanism refinements are only applicable to closed-loop neural control.

In the neural encoding model category are four enhancements. (1) We use the Cartesian coordinates of hand acceleration and a novel acceleration magnitude. (2) We include a novel multiplicative hand position and hand velocity interaction term in the encoding model. (3) We include the target position and a novel target-cursor distance term in the encoding model. (4) We include the recent spiking history (i.e., spike count in the previous time bin) of the entire recorded population in the encoding model.

For the control mechanism, we have added three ideas: (1) we use a mixture of decoded position and decoded velocity to derive the new cursor position (Homer M. et al., 2013). (2) We use a novel probabilistic threshold for velocity, which detects when the user is trying to move in a more principled way than a simple threshold on velocity. (3) We use predicted future kinematics to drive the cursor, which is intended to improve the responsiveness of the decoder.

These refinements are specified below and discussed in the discussion section. Table S1 in the Supplementary Materials provides an overview of which refinements are used during offline reconstructions and closed-loop decoding.

### State variables and neural tuning model

The filter's state variables are the variables to be decoded, but can also include other variables which may improve the accuracy of the neural encoding model. In the standard position-velocity Kalman filter, the desired position and velocity in the x and y axis are the state variables (for a 2D task). In the UKF1, the state has multiple taps of position and velocity. That is, at time *t* (discrete index which counts bins), the UKF1's state variables are the position and velocity at time *t, t* + 1, *t* + 2, *t* + 3, *t* + 4, *t* + 5, *t* − 1, *t* − 2, *t* − 3, and *t* − 4, for a total of 10 taps. These taps include estimates of “future” values of the kinematics as well as “past” values, relative to the current time *t*. By including future taps and past taps, the UKF1 is able to model neural tuning at multiple time offsets simultaneously. Since the task is two-dimensional, the number of state variables is 2 (dimensions) × 2 (position, velocity) × 10 (taps) = 40.

In the UKF2 decoder, hand acceleration and target position are added to the state variables. The target position is a decoded variable and not required as input to the decoder. The number of taps is reduced to five. The number of state variables is thus 2 (dimensions) × 4 (cursor position, velocity, acceleration; target position) × 5 (taps) = 40.

The neural encoding model or tuning model is the observation model of the (unscented) Kalman filter when it is used as a decoder. It is a generative model that predicts the binned spike count (instantaneous firing rate) given the state variables' values. The neural encoding model works in one direction, while the decoder, which wraps around it, works in the other direction by “inverting” the encoding model. To decode a variable, it must be present as a feature in the encoding model and as a state variable in the Kalman filter; however, not all encoding model features are decoded by the decoder (e.g., spiking history), and not all state variables are output by the decoder to control the cursor. The encoding model of the position-velocity Kalman filter is:

(1) $$\begin{array}{l} {fr}_{i,t}^{\text{PVKalman}}\approx c\cdot {px}_{t}+c\cdot {py}_{t}+c\cdot {vx}_{t}+c\cdot {vy}_{t}, \end{array}$$

where *fr*_*i, t*_ is the (mean subtracted) firing rate of unit *i* at time *t*, every instance of *c* is a different coefficient fitted from training data, *px*_*t*_ and *py*_*t*_ are the *x*-axis and *y*-axis positions at time *t*, respectively, and *vx*_*t*_ and *vy*_*t*_ are the *x*-axis and *y*-axis velocities at time *t*, respectively.

In the UKF1, the encoding model is:

(2) $$\begin{array}{rcl} {fr}_{i,t}^{\text{UKF}1} & \approx & \text{position}\left(t+5\right)+\text{velocity}\left(t+5\right)+\text{position}\left(t+4\right)\\ +\text{velocity}\left(t+4\right)+\cdots +\text{position}\left(t-4\right)\\ +\text{velocity}\left(t-4\right). \end{array}$$

For clarity, we have broken down the model to contributions from different kinematic features:

(3) $$\begin{array}{l} \text{position}\left(k\right)=c\cdot {px}_{k}+c\cdot {py}_{k}+c\sqrt{{px}_{k}^{2}+{py}_{k}^{2}}, \end{array}$$

(4) $$\begin{array}{l} \text{velocity}\left(k\right)=c\cdot {vx}_{k}+c\cdot {vy}_{k}+c\sqrt{{vx}_{k}^{2}+{vy}_{k}^{2}}. \end{array}$$

Again, to reduce notational clutter, every instance of c is a different coefficient (total 60).

Combining the model of UKF1 and the above outlined encoding model enhancements, and reducing the number of taps to five, we obtain the encoding model of the UKF2:

(5) $$\begin{array}{rcl} {fr}_{i,t}^{\text{UKF}2} & \approx & \text{position}\left(t+2\right)+\text{velocity}\left(t+2\right)+\text{acceleration}\left(t+2\right)\\ +\text{ interaction}\left(t+2\right)+\text{target}\left(t+2\right)+\ldots \\ +\text{ position}\left(t-2\right)+\text{velocity}\left(t-2\right)+\text{acceleration}\left(t-2\right)\\ +\text{ interaction}\left(t-2\right)+\text{target}\left(t-2\right)+\text{spiking}\left(t-1\right), \end{array}$$

(6) $$\begin{array}{rcl} \text{acceleration}\left(k\right) & = & c\cdot {ax}_{k}+c\cdot {ay}_{k}+c\sqrt{{ax}_{k}^{2}+{ay}_{k}^{2}}, \end{array}$$

(7) $$\begin{array}{rcl} \text{interaction}\left(k\right) & = & c\cdot \left({px}_{k}\cdot {vx}_{k}\right)+c\cdot \left({py}_{k}\cdot {vy}_{k}\right), \end{array}$$

(8) $$\begin{array}{rcl} \text{target}\left(k\right) & = & c\cdot {tx}_{k}+c\cdot {ty}_{k}\\ +\;c\sqrt{{\left({tx}_{k}-{px}_{k}\right)}^{2}+{\left({ty}_{k}-{py}_{k}\right)}^{2}}, \end{array}$$

(9) $$\begin{array}{rcl} \text{spiking}\left(k\right) & = & c\cdot {fr}_{1,k}^{\text{actual}}+c\cdot {fr}_{2,k}^{\text{actual}}+\cdots +c\cdot {fr}_{n,k}^{\text{actual}}, \end{array}$$

where *ax*_*t*_ and *ay*_*t*_ are the *x*-axis and *y*-axis hand accelerations at time *t*, respectively, *tx*_*t*_ and *ty*_*t*_ are the *x*-axis and *y*-axis target positions at time *t*, respectively, n is the number of units, and as before every instance of c is a different coefficient (total 70 + *n*). Note that our use of target position in the encoding model includes a target-to-cursor distance term. The five taps of kinematic features are followed by spiking history terms for the entire population of *n* units [in spiking(*t* − 1)]. This spiking history is the spike count in the previous bin for the entire population.

### Mixing position and velocity outputs

Now we describe the first of the control mechanism refinements. These refinements operate on the output of the unscented Kalman filter. The outputs, or “decoded” variables, are the means of the state variables of the unscented Kalman filter after it has performed its filtering operations. These decoded variables are processed further by the methods described below to get the final on-screen cursor position.

Instead of using only the decoded position to control the cursor as in UKF1, for the UKF2, both the decoded velocity and the decoded position are used to update the cursor during closed-loop operation. The two inputs are mixed together using a mixing coefficient (Homer M. et al., 2013):

(10) $$\begin{array}{l} {\bar{x}}_{t}={\bar{x}}_{t\;-\;1}+c_{m}\cdot \text{dt}\cdot {\bar{v}}_{t}+\left(1-c_{m}\right)\text{dt}\frac{\left||{\bar{v}}_{t}|\right|}{\left||{\bar{e}}_{t}|\right|}{\bar{e}}_{t}, \end{array}$$

(11) $$\begin{array}{l} {\bar{e}}_{t}={\bar{p}}_{t}-{\bar{x}}_{t-1}, \end{array}$$

where ${\bar{x}}_{t}$ is the vector of on-screen cursor position at time *t*, *c*_*m*_ is the mixing coefficient (0.5), dt is the delta time between time steps (50 ms), ${\bar{v}}_{t}$ is the decoded velocity vector at time *t* [i.e., ${\bar{v}}_{t}=\left({vx}_{t\;+\;2},\;{vy}_{t\;+\;2}\right)$, the +2 is due to use of future predictions, described below], ${\bar{e}}_{t}$ is an intermediate variable representing the difference between the decoded position and previous on-screen cursor location, and ${\bar{p}}_{t}$ is the decoded position vector at time *t* [i.e., ${\bar{p}}_{t}=\left({px}_{t\;+\;2},\;{py}_{t\;+\;2}\right)]$. One way to interpret these equations is that the decoded velocity magnitude acts as a gate or limit for the amount that the decoded position can affect the cursor. We did not use this feature in offline reconstructions and analysis. Note this control mechanism refinement is orthogonal to the position-velocity interaction refinement in the encoding model.

### Movement thresholding

We add a probability-based mechanism to help the user stop the cursor during closed-loop control (only) which applies a threshold on the decoded velocity. Since the Kalman filter provides a covariance estimate for the velocity state variable, we can perform a check using the decoded mean and covariance values that allows us to place a threshold in terms of false positive rate. Since the distribution of the state variables is assumed to be multivariate normal, the x-axis and y-axis velocity values together form a vector that has a two-dimensional multivariate normal distribution. Given the mean vector ($\bar{v}$) and covariance matrix (*C*_*v*_) of this distribution, we can compute the statistic:

(12) $$\begin{array}{l} X={\bar{v}}^{\text{T}}C_{\text{v}}^{-1}\bar{v},\;X\sim \;X^{2}\left(2\right) \end{array}$$

*X* has a chi-squared distribution with 2 degrees of freedom. We can consult the cumulative distribution function of the chi-squared distribution to test if the velocity is significantly different from zero with a given α-value.

During decoding, the decoded velocity outputted by the UKF2 is tested using this method. If the null hypothesis is rejected, i.e., the user is deemed to want to move the cursor, unscented Kalman filtering proceeds as normal and the decoded position and velocity are mixed as described in the previous section. Otherwise, the cursor is not moved (skipping mixing of position and velocity) and in the next iteration, instead of adding the velocity to the position in the execution of the transition model, we do not modify the position. Note that this procedure does not set the velocity variable in the state to zero. If we were to do that, accelerating from zero velocity might be difficult: if the velocity increases from zero to a small value, but does not pass the threshold, it is then set to zero again. By not editing the velocity in the state, we allow it to build up over time to exceed the probabilistic threshold. In our experience, a *p*-value of around 0.3 worked well, and we used values in the range 0.1–0.5 in closed-loop experiments, varying with session. We did not use this enhancement in offline reconstructions and analysis.

### Use of future predictions

During closed-loop neural control, we used filter predictions of future intentions to drive the cursor for the UKF2. The UKF1 included multiple taps in its state. This allowed the filter's observation model to capture tuning relationships between kinematic variables and neural firing rates at multiple time offsets simultaneously. A concrete example is that the x-axis velocity at time bins *t* − 4, *t* − 3…*t* + 5 are all included in the function that models the spike count at time bin *t*. However, for the UKF1, the position in the filter state corresponding to time *t* is used to control the cursor when the filter is processing neural activity at time *t*, i.e., there is no temporal offset.

We keep five taps of kinematic variables in the state of the UKF2, *t* − 2, *t* − 1, *t, t* + 1, *t* + 2, where *t* is the time of the bin of neural activity the filter is currently processing. We use the estimated kinematics at the *t* + 2 tap to control the cursor during closed-loop neural control of the cursor. With our bin size of 50 ms, this amounts to a temporal offset of 100 ms. This offset is in the causal direction, i.e., compatible with the notion that neural activity encodes for movement occurring 100 ms later. For offline reconstructions and analysis, the UKF2 used the zero offset temporal tap (*t*), otherwise we would introduce error due to temporal misalignment.

### Decoder comparison

We compared the improved unscented Kalman filter based decoder (UKF2) with the previously published unscented Kalman filter based decoder (UKF1). The settings for UKF1 differ from Li et al. (2009) in several ways. First, the bin size was 50 ms instead of 100 ms, so as to be easily comparable to UKF2 using the same training data. Second, in offline reconstructions the UKF1 transition model used one time tap of kinematics to estimate the future-most tap of kinematics, whereas, in our previous work, the UKF1 estimate was based on all (10) taps in the state. This change improved filter stability: when all 10 taps are used, forming a 10th order autoregressive model, the fitted transition model was more likely to cause filter instability. In closed-loop experiments, the transition model was pre-designed around physical laws of motion, as described in the Model Fitting section. This contrasts to our previous work, where the transition model was always fit to data. We made this change so that all three decoders would use transition models based on physical laws of motion, since transition model design is not the focus of this study.

We also compare with a Kalman filter which includes position and velocity in its state space (position-velocity Kalman filter). During closed-loop decoding, this Kalman filter used two refinements developed by other researchers: modeling position as a feedback signal (Gilja et al., 2012) and using intention estimates to fit observation model parameters (Gilja et al., 2012; Fan et al., 2014), which makes it equivalent to the FIT Kalman filter (Fan et al., 2014). We do not use the re-training paradigm of the ReFIT Kalman filter (Gilja et al., 2012), because we needed to keep the training data for all of the tested decoders the same to achieve a fair comparison. Moreover, adding a re-training phase to the experiment protocol would allow more time to practice using the Kalman filter decoder, and inject additional variation in animal behavior. Since Fan et al. (2014) reported that intention estimation applied to initial training data had comparable benefits as retraining with intention estimation, we opted to use intention estimation on initial training data in our experimental protocol. See the Supplementary Materials for the implementation of the two refinements.

A brief description of the Kalman and unscented Kalman filters and a table summarizing the compared decoders can be found in the Supplementary Materials.

### Model fitting

We fitted the encoding models of all decoders using the same training portion of each session. This data consisted of population binned spike counts and simultaneously recorded cursor positions (equivalent to transformed hand positions), velocities, accelerations, and target positions, if applicable. The encoding models included terms which were non-linear in the state variables, but the coefficients for them can be fitted in a linear regression since the non-linear terms can be pre-calculated as features. We used Tikhonov regularized linear regression (ridge regression) to fit the coefficients of the models, with automatic finding of the best ridge parameter. The parameter fitting procedure is very similar to the one in Li et al. (2009), except for the details of the ridge parameter selection scheme (see Supplementary Materials). We used this parameter fitting procedure to fit the coefficients of the encoding models for all analysis.

In offline reconstructions, the transition model of all three decoders were fitted to training data in the same way as Li et al. (2009), except that we used the newer scheme described in the Supplementary Materials for choosing the ridge regression parameter. Note that this means the target position is decoded, but otherwise does not directly affect the other variables.

In closed-loop neural control, the transition models of all three decoders were set to be similar to the equations describing physical laws. For the Kalman filter:

(13) $$\begin{array}{l} {\bar{p}}_{t\;+\;1}={\bar{p}}_{t}+{\bar{v}}_{t}\cdot \text{dt}, \end{array}$$

(14) $$\begin{array}{l} {\bar{v}}_{t\;+\;1}={c_{\text{v}}\cdot \bar{v}}_{t}+{\bar{\epsilon }}_{\text{v}}, \end{array}$$

where ${\bar{p}}_{t}$ and ${\bar{v}}_{t}$ are the position and velocity vectors at time *t*, respectively, c_v_ is the coefficient representing friction (0.85), dt is delta time between filter iterations, i.e., the bin width (50 ms), and ${\bar{\epsilon }}_{\text{v}}$ is the random noise on the velocity (details below for fitting procedure). The position lacks a noise term since we used the position-as-feedback scheme. The friction term gives the cursor a virtual mass, which makes it easier to control.

The transition model for the UKF1 is slightly different since it has multiple taps in the state space. We set the leading tap similar to above:

(15) $$\begin{array}{l} {\bar{p}}_{t\;+\;6}={\bar{p}}_{t\;+\;5}+{\bar{v}}_{t\;+\;5}\cdot \text{dt}+{\bar{\epsilon }}_{\text{p}}, \end{array}$$

(16) $$\begin{array}{l} {\bar{v}}_{t\;+\;6}={c_{\text{v}}\cdot \bar{v}}_{t\;+\;5}+{\bar{\epsilon }}_{\text{v}}. \end{array}$$

Note that position also has a random noise term. For the other taps, values are propagated through time without change.

The UKF2 model includes acceleration, thus we change our equation for velocity and include an equation for acceleration. Acceleration is constant except for a decay coefficient and random noise. Furthermore, target position is included as a constant value modified by random noise. Target position attracts the cursor by affecting acceleration during closed loop control. The UKF2 transition model for the leading tap is:

(17) $$\begin{array}{l} {\bar{p}}_{t\;+\;3}={\bar{p}}_{t\;+\;2}+{\bar{v}}_{t\;+\;2}\cdot \text{dt}+{\bar{\epsilon }}_{\text{p}}, \end{array}$$

(18) $$\begin{array}{l} {\bar{v}}_{t\;+\;3}={c_{\text{v}}\cdot \bar{v}}_{t\;+\;2}+{\bar{a}}_{t\;+\;2}\cdot \text{dt}+{\bar{\epsilon }}_{\text{v}}, \end{array}$$

(19) $$\begin{array}{l} {\bar{a}}_{t\;+\;3}=c_{\text{a}}{\cdot \bar{a}}_{t\;+\;2}+c_{\text{g}}\left({\bar{g}}_{t\;+\;2}-{\bar{p}}_{t\;+\;2}\right)/{\text{dt}}^{2}+{\bar{\epsilon }}_{\text{a}}, \end{array}$$

(20) $$\begin{array}{l} {\bar{g}}_{t\;+\;3}={\bar{g}}_{t\;+\;2}+{\bar{\epsilon }}_{\text{g}}, \end{array}$$

where ${\bar{a}}_{t}$ and ${\bar{g}}_{t}$ are the vectors of acceleration and target position at time *t*, respectively, *c*_*a*_ is the coefficient representing acceleration decay (0.75), and *c*_*g*_ is the gain on the attraction effect of the target position (0.01). We add acceleration decay to prevent an error in acceleration decoding from affecting decoded output for an unlimited duration. For the other taps, values are propagated through time without change. The values of the constants were picked to be similar to typical values seen when fitting transition models to training data in our preliminary analysis. We did this so that the values would be similar to fitted values, but do not change per closed-loop recording session.

The transition model noise covariance matrices, which describe the joint distribution of the noise terms (${\bar{\epsilon }}_{\text{p}},\;{\bar{\epsilon }}_{\text{v}},\;{\bar{\epsilon }}_{\text{a}}$, and $\;{\bar{\epsilon }}_{\text{g}}$) were fitted under the above specified models. That is, the above physics-based models were used to predict the variables (using one time step as the input and having the next time step be the desired output), and the sample covariance matrix of the prediction residuals was used as the transition model noise covariance matrix. Note that, for the FIT Kalman filter, we set the positional noise's variance and covariance entries to zero to achieve the position-as-feedback enhancement of Gilja et al. (2012).

### Experiment procedure

We first compared the performance of decoders in making offline reconstructions. For this, we used portions of sessions where the monkey controlled the cursor with its hand. We reconstructed the cursor movements with each decoder and measured the accuracy of the reconstructions vs. the actual cursor movements. The data was divided into training and testing portions. We ignored the first 30 s of data to avoid transient problems, used the subsequent 10 min for the training portion, and set aside the remainder as the testing portion. If the session had less than 12.5 min of hand control data, we used 5 min of data for training (the shortest session had 8 min).

We measured accuracy of reconstructions by computing the correlation coefficient (CC) and signal to noise ratio (SNR, see below for equation). CC or SNR for each Cartesian axis' position and velocity were computed separately and combined by averaging. For SNR, the arithmetic mean in decibels was calculated. We did not use mixing of position and velocity (Homer M. et al., 2013) or the position-as-feedback scheme (Gilja et al., 2012) in reconstructions, as those are designed for closed-loop control.

We compared the encoding accuracy of the UKF2 encoding model with the encoding model of the UKF1 and the position-velocity linear model of the Kalman filter decoder. To do this, we again used portions of sessions where the monkey controlled the cursor with its hand. Since the number of parameters differs substantially between models, a comparison of model fit may be biased toward the more complex model. Therefore, we compared the ability of the models to predict binned spike counts on testing data that was not used to fit model parameters. Model predictions on separate testing data are unbiased toward more complex models, since prediction accuracy reflects generalization accuracy.

We split the data into training and testing portions using a two-fold cross-validation procedure. We used the training portions to fit the parameters of the encoding models. Then we tested the encoding models by providing kinematics data (and past spike counts, if applicable) and then predicting spike counts. We compared the predicted spike counts with the actual spike counts in the testing portion and calculated accuracy using the correlation coefficient or the signal-to-noise ratio (SNR). We repeated this procedure, switching training and testing data, and averaged results between the two repetitions. For model predictions, we only used one tap of kinematics for all models, since our previous work has already shown the advantage of using multiple taps (Li et al., 2009) and that is not the focus of this study.

Finally, we compared the ability of monkeys to use the decoders, in turn, to control a cursor in closed-loop neural control. All the decoders' parameters were fitted on the same initial training data (10 min.), collected at the beginning of each session when the monkey used its hand to control the cursor. The order of use of the decoders was shuffled across sessions to average out order effects. In each session (day), each decoder was used for 10 min, with the first 5 min for familiarization and the last 5 min used for accuracy calculation. During neural control of cursor, the monkey continued to manipulate the joystick, even though it was disconnected (i.e., brain control with hand movements).

When analyzing the closed-loop performance data, we calculated fraction of targets acquired, movement time, and Fitts's Law bit rate (Gilja et al., 2012). Shorter movement durations meant the monkey could move and hold the cursor in the target faster, which reflects better controllability. Since we kept target sizes and reach distances constant, the Fitts's Law bit rate was monotonic with the movement time. For fraction of targets acquired, we included acquisition of the center target as well as peripheral targets. We only considered movements from the center to the periphery for movement time and Fitts's Law bit rate. Since the monkey sometimes paused during performance of the task due to lack of motivation or distraction, failure in the task may occur due to inactivity. We observed that, when the monkey was actively participating, the percentage of successful trials was high (>90%). Thus, to eliminate failures due to inactivity from the time and rate calculations, which are confounds not related to decoder performance, we only analyzed the movement time and Fitts's Law bit rate of successful center to peripheral trials which followed successful acquisition of the center target.

The SNR, in decibels, was calculated by:

(21) $$\begin{array}{l} {SNR}_{dB}\;=\;10\cdot {log}_{10}\left(\frac{{Var}_{s}}{MSE}\right), \end{array}$$

where *Var*_*s*_ is the variance of the desired signal, e.g., recorded position during reconstructions or measured spike counts during encoding model analysis, and MSE is the mean squared error between the desired signal and decoded value, e.g., reconstructed kinematics or predicted spike counts. The SNR can be seen as a normalized, inverted, and log-transformed mean squared error. Unlike the CC, SNR does not saturate. It also detects scale and offset errors which CC cannot. We believe it is better than the mean squared error because it is normalized and thus more comparable across experimental setups, does not saturate, and naturally increases with quality.

For statistical analysis, we used two-factor analysis of variance (decoder × session, or model × unit) with single replication, and we focus on the decoder and model differences. *Post-hoc* multiple comparison testing was conducted with two-tailed paired *t*-tests with *p*-values corrected by the Holm-Bonferroni method. All testing used a significance level of α = 0.05.

## Results

### Offline reconstructions

**Figure 3:**
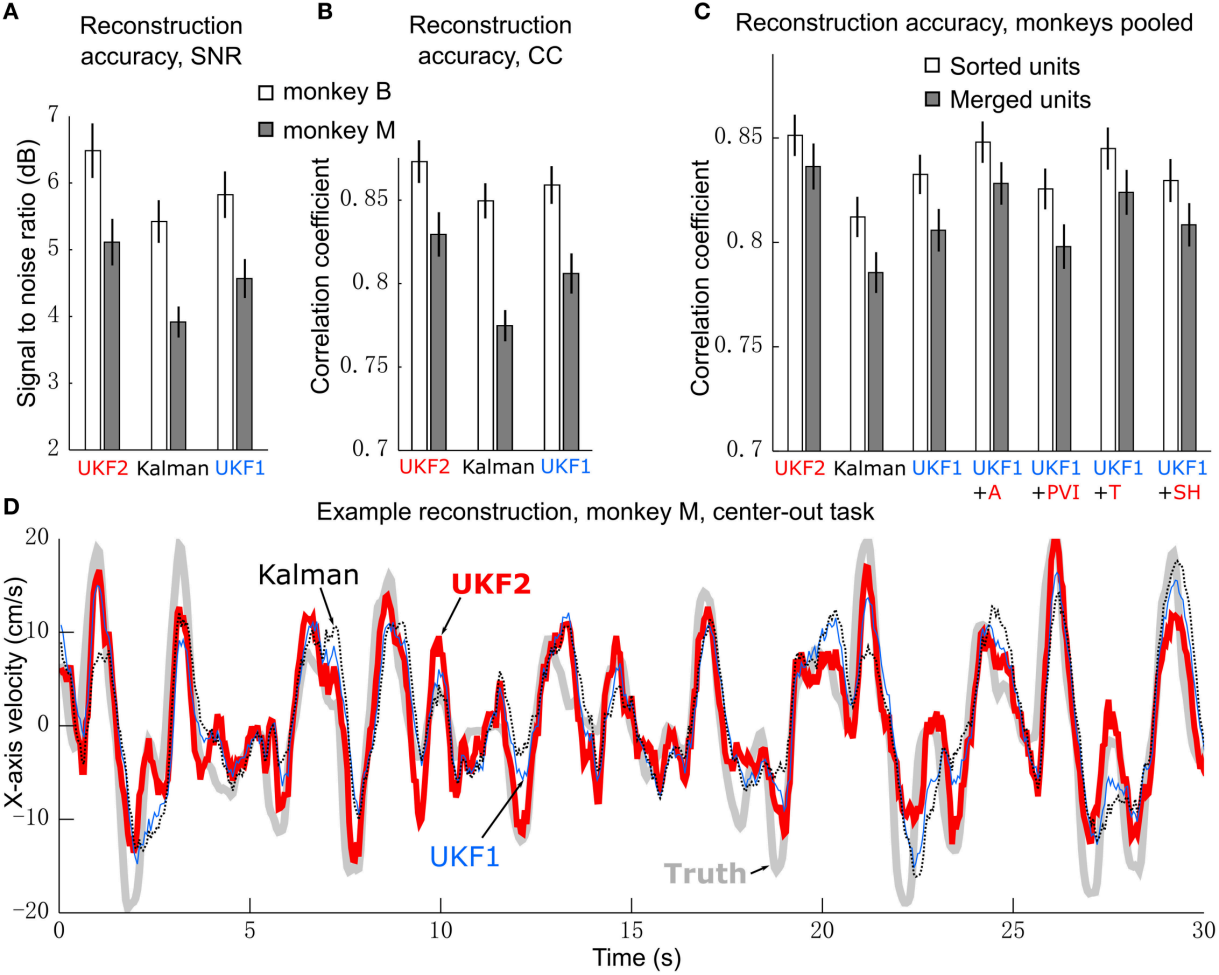
**Offline reconstruction accuracy. (A)** Mean ± SEM of signal-to-noise ratios. **(B)** Mean±SEM of correlation coefficients. **(C)** Reconstruction accuracy when pooling data from two monkeys. White bars show accuracy when using spike sorting. Gray bars show accuracy when using unsorted spikes derived by merging all sorted units on each channel. Right side bars show UKF1 augmented with each of: +A, acceleration; +PVI, position-velocity interaction; +T, target; +SH, spiking history of population. **(D)** Example reconstruction of x-axis velocity from one session of monkey M.

We compared the ability of the decoders to reconstruct hand-controlled cursor trajectories. We analyzed 16 sessions from monkey B, recorded 24–97 days post-implant, and 16 sessions from monkey M, recorded 17–162 days post-implant. Hand-controlled portions of these sessions ranged from 8 to 73 min in length, with mean 27.2 min. For each session, reconstructions were performed by using up to 10 min to fit parameters of models and then reconstructing the remainder of the hand-controlled portion of the session (see Experiment Procedure). The results are summarized in Table 1 and graphed in Figure 3.

**Table 1:** **Offline reconstruction accuracy**.

**Mean ± SEM**	**CC, monkey B**	**CC, monkey M**	**CC, pooled**	**CC, pooled, merged units**	**SNR (dB), monkey B**	**SNR (dB), monkey M**	**SNR (dB), pooled**	**SNR (dB), pooled, merged units**
UKF2	0.873 ± 0.013	0.829 ± 0.013	0.851 ± 0.010	0.836 ± 0.011	6.484 ± 0.409	5.113 ± 0.349	5.799 ± 0.292	5.295 ± 0.266
Kalman	0.850 ± 0.011	0.775 ± 0.009	0.812 ± 0.010	0.786 ± 0.010	5.423 ± 0.320	3.917 ± 0.233	4.670 ± 0.237	4.008 ± 0.200
UKF1	0.859 ± 0.011	0.806 ± 0.012	0.833 ± 0.009	0.806 ± 0.010	5.824 ± 0.349	4.568 ± 0.292	5.196 ± 0.251	4.488 ± 0.226
UKF1+A	0.868 ± 0.010	0.828 ± 0.016	0.848 ± 0.010	0.828 ± 0.010	6.019 ± 0.370	5.009 ± 0.389	5.514 ± 0.279	4.854 ± 0.249
UKF1+PVI	0.852 ± 0.012	0.799 ± 0.012	0.826 ± 0.010	0.798 ± 0.011	5.663 ± 0.352	4.437 ± 0.295	5.050 ± 0.251	4.349 ± 0.230
UKF1+T	0.871 ± 0.010	0.819 ± 0.015	0.845 ± 0.010	0.824 ± 0.011	6.193 ± 0.361	4.839 ± 0.364	5.516 ± 0.280	4.847 ± 0.253
UKF1+SH	0.865 ± 0.013	0.794 ± 0.010	0.830 ± 0.010	0.808 ± 0.010	6.160 ± 0.379	4.422 ± 0.231	5.291 ± 0.268	4.712 ± 0.228

*A, acceleration; PVI, position-velocity interaction; T, target; SH, spiking history of population; Pooled, combining data from two monkeys; Merged units, undoing spike sorting by merging units in each channel*.

We calculated the SNR of the trajectory reconstructions for each decoder (Figure 3A). ANOVA found a main effect of decoder [monkey B: *F*_(2, 15)_ = 27.03, *p* = 1.94 × 10^−7^, monkey M: *F*_(2, 15)_ = 42, *p* = 2.01 × 10^−9^] and *post-hoc* testing showed that all three decoders were significantly different from each other (monkey B: all corrected *p* < 0.0024, monkey M: all corrected *p* < 0.00007). We also quantified accuracy in terms of correlation coefficient (CC) (Figure 3B). ANOVA found a main effect of decoder [monkey B: *F*_(2, 15)_ = 15.59, *p* = 2.28 × 10^−5^, monkey M: *F*_(2, 15)_ = 53.86, *p* = 1.18 × 10^−10^] and *post-hoc* testing showed that all three decoders were significantly different from each other (monkey B: all corrected *p* < 0.015, monkey M: all corrected *p* < 0.00002). The UKF2 reconstructed most accurately among the three decoders.

We pooled data from both monkeys and analyzed the contributions of each of the different encoding model enhancements used in the UKF2 decoder, in terms of CC (Figure 3C, white bars). To do this, we added to the UKF1 model each of the model enhancements in turn: acceleration (+A), position-velocity interactions (+PVI), target position (+T), which includes the target-to-cursor distance term, and spiking history of the population (+SH). For the pooled data, ANOVA found a main effect of decoder [*F*_(6, 31)_ = 29.32, *p* < 10^−10^] and *post-hoc* comparisons showed that UKF2 was significantly more accurate than KF (corrected *p* < 10^−6^) and UKF1 (corrected *p* = 6 × 10^−6^) and UKF1 was significantly more accurate than KF (corrected *p* = 0.000014). In terms of feature contributions, UKF1+A was significantly more accurate than UKF1 alone (corrected *p* = 0.00006). UKF1+PVI was significantly less accurate than UKF1 alone (corrected *p* = 0.0035). UKF1+T was significantly more accurate than UKF1 alone (corrected *p* = 0.00092). UKF1+SH was not significantly different from UKF1 (corrected *p* = 0.39). UKF2 was significantly more accurate than UKF1 augmented with PVI (corrected *p* = 3 × 10^−6^) and SH (corrected *p* = 0.000014). Significance testing results when using SNR values were similar [main effect of decoder, *F*_(6, 31)_ = 33.49, *p* < 10^−10^], except that UKF2 was also significantly better than UKF1+A (corrected *p* = 0.0019) and UKF1+T (corrected *p* = 0.00081). Comparing the contribution of individual features in terms of CC, UKF1+A was better than UKF1+PVI (corrected *p* = 0.000013) and UKF1+SH (corrected *p* = 0.0011), and UKF1+T was better than UKF1+PVI (corrected *p* = 0.00023) and UKF1+SH (corrected *p* = 0.0017). These comparisons indicate acceleration and target tuning contributed the most to reconstruction accuracy.

It was concerning that the UKF1+PVI reconstructed less accurately than UKF1 alone. When we examined behavioral tasks separately, we found that for the pursuit task (both variants combined), UKF1+PVI (0.831 ± 0.026, mean CC±SEM) was nominally higher than UKF1 alone (0.829 ± 0.025), though the difference was not significant (two-tailed paired *t*-test, uncorrected, *n* = 6, *p* = 0.103). We think this is due to the more thorough sampling of the space of possible position/velocity combinations seen in the pursuit task data.

Accuracy for monkey M was generally poorer, and upon examining fitted model parameters, we suspected that the spiking history features were not fitted as well for monkey M. Thus, we examined the contribution of spiking history per monkey. For monkey B, in terms of CC, UKF1+SH was significantly more accurate than UKF1 alone (two-tailed paired *t*-test, uncorrected, *n* = 16, *p* = 0.04995), but for monkey M, UKF1+SH was significantly less accurate than UKF1 alone (two-tailed paired *t*-test, uncorrected, *n* = 16, *p* = 0.0001). The significance testing results were the same for SNR.

As described in the methods, we spike sorted aggressively, which resulted in a large number of multiunits. We wondered if our aggressive spike sorting affected the observed trends. Thus, we tried performing reconstructions using unsorted spiking data. We merged all units on each channel, undoing the process of spike sorting. Note that this is different from using all threshold crossings, since waveforms which crossed the threshold but did not match any unit's template are excluded. The reconstruction results from merged units are shown by the gray bars in Figure 3C. The mean accuracy when using merged units was significantly and substantially worse than the mean accuracy when using sorted units under every decoder variant (two-tailed paired *t*-tests, all corrected *p* < 0.000035). ANOVA (merged decoders x session) found a main effect of decoder [*F*_(6, 31)_ = 34,31, *p* < 10^−10^]. The trends in accuracy among UKF2, KF, UKF1 and when enhancements are individually added to UKF1 for merged units were similar to the trends from sorted units (except that UKF2 was significantly better than UKF1+T, corrected *p* = 0.0028), confirming that our aggressive spike sorting did not influence the trends we observed.

We illustrate sample reconstructions of the UKF2, position-velocity Kalman filter, and UKF1 in Figure 3D. In this panel, we show the 30 s of reconstructed x-axis velocity vs. time from one center-out session of monkey M. We can see that the UKF2 reconstruction follows the cursor velocity better at several peaks and valleys, though there are also instances where UKF2's reconstruction is farthest from the true value.

### Encoding model predictions

**Figure 4:**
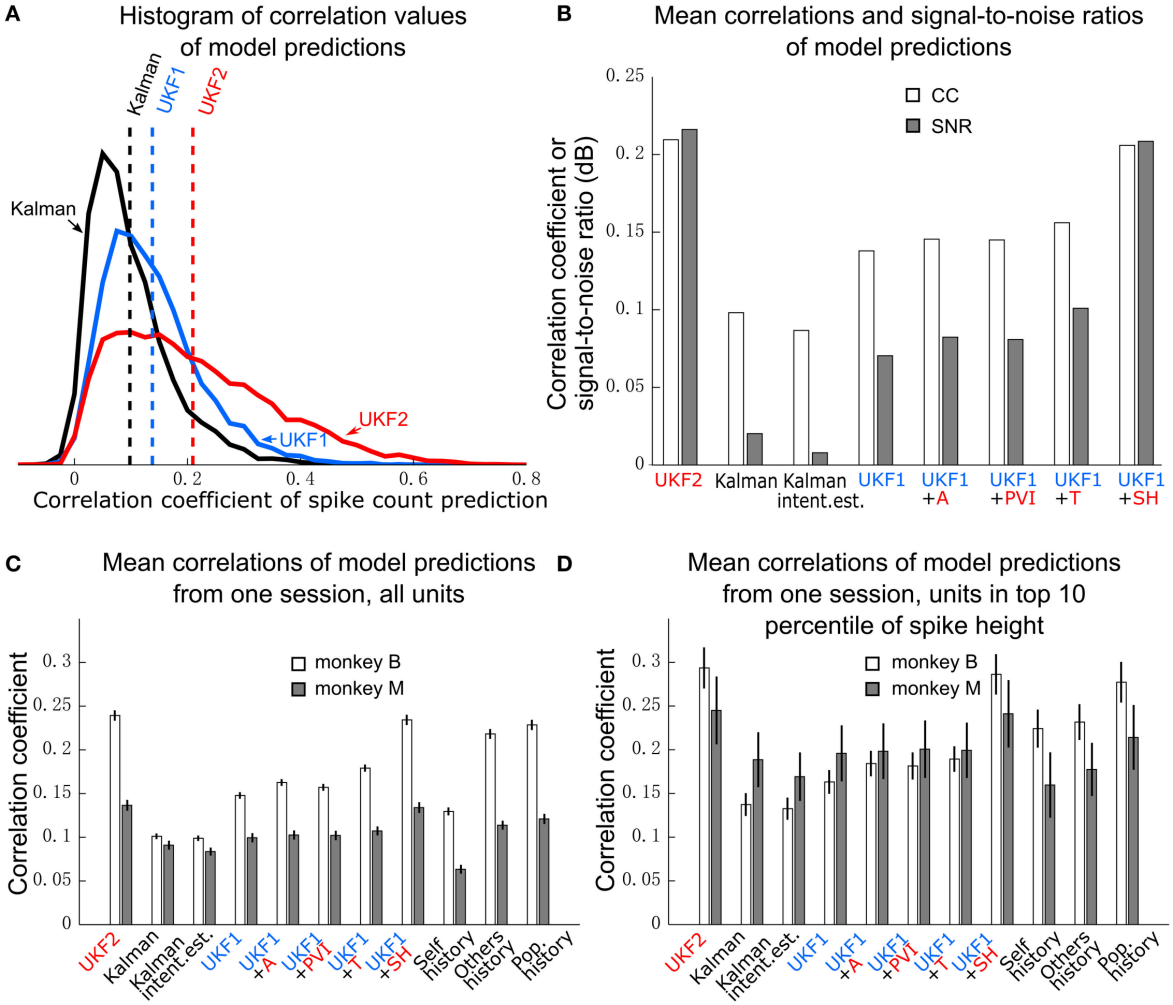
**Encoding model prediction accuracy. (A)** Histogram of spike count prediction accuracies measured by correlation coefficient. Dashed vertical lines indicate means for each encoding model. **(B)** Mean CC and SNR of each encoding model, including UKF1 augmented with each of: +A, acceleration; +PVI, position-velocity interaction; +T, target; +SH, spiking history of population. SEM was not calculated since data likely includes repeated observations of neurons. **(C)** Mean±SEM of CC from one session of each monkey when using all sorted units. **(D)** Mean ± SEM of CC from one session of each monkey when using units with mean spike height (peak to trough) in the top 10 percentile of all units in the session.

We next compared the encoding model of the UKF2 with that of UKF1 and the position-velocity Kalman filter. The purpose of this is to better understand what motor cortical neurons encode and to explain the improvements in decoding accuracy—it would be unsatisfying if the source of decoding improvements were unknown—and not as evidence for better decoding accuracy. We analyzed the same 16 sessions from monkey B and 16 sessions from monkey M, which together had a total of 8582 single units and multiunits. These units represent a far smaller number of distinct neurons, since electrodes usually record the same neurons between sessions. Due to this, we also conducted analysis on a single session from each monkey, so as to obtain unique units for significance testing. The results are summarized in Table 2 and graphed in Figure 4.

**Table 2:** **Encoding model prediction accuracy**.

**Mean ± SEM**	**CC, pooled**	**CC, monkey B, one session**	**CC, monkey M, one session**	**CC, monkey B, one session, top 10 percentile units**	**CC, monkey M, one session, top 10 percentile units**	**SNR(dB), pooled**	**SNR(dB), monkey B, one session**	**SNR(dB), monkey M, one session**
UKF2	0.210 ± 0.002	0.239 ± 0.006	0.137 ± 0.006	0.294 ± 0.024	0.245 ± 0.039	0.216 ± 0.005	0.285 ± 0.019	0.075 ± 0.017
Kalman	0.098 ± 0.001	0.101 ± 0.003	0.091 ± 0.005	0.137 ± 0.013	0.189 ± 0.031	0.020 ± 0.002	0.050 ± 0.004	0.033 ± 0.010
Kalman intention estimation	0.087 ± 0.001	0.099 ± 0.003	0.084 ± 0.005	0.133 ± 0.013	0.169 ± 0.028	0.008 ± 0.002	0.047 ± 0.004	0.023 ± 0.008
UKF1	0.138 ± 0.001	0.148 ± 0.004	0.099 ± 0.005	0.163 ± 0.014	0.196 ± 0.032	0.070 ± 0.003	0.109 ± 0.007	0.041 ± 0.010
UKF1+A	0.145 ± 0.001	0.163 ± 0.004	0.103 ± 0.005	0.184 ± 0.015	0.198 ± 0.032	0.082 ± 0.003	0.133 ± 0.007	0.043 ± 0.010
UKF1+PVI	0.145 ± 0.001	0.157 ± 0.004	0.102 ± 0.005	0.181 ± 0.016	0.201 ± 0.033	0.081 ± 0.003	0.125 ± 0.007	0.044 ± 0.011
UKF1+T	0.156 ± 0.001	0.179 ± 0.004	0.107 ± 0.005	0.189 ± 0.015	0.199 ± 0.032	0.101 ± 0.003	0.160 ± 0.008	0.046 ± 0.010
UKF1+SH	0.206 ± 0.001	0.234 ± 0.006	0.134 ± 0.006	0.286 ± 0.023	0.241 ± 0.039	0.208 ± 0.005	0.272 ± 0.018	0.071 ± 0.016
Self history	–	0.130 ± 0.005	0.063 ± 0.005	0.224 ± 0.022	0.160 ± 0.037	–	0.104 ± 0.010	0.024 ± 0.011
Others history	–	0.218 ± 0.006	0.114 ± 0.005	0.232 ± 0.021	0.177 ± 0.030	–	0.225 ± 0.016	0.034 ± 0.011
Population history	–	0.229 ± 0.006	0.121 ± 0.006	0.277 ± 0.023	0.214 ± 0.037	–	0.259 ± 0.018	0.054 ± 0.015

*Kalman, linear encoding model of the position-velocity Kalman filter decoder; Kalman intention estimation, training and testing hand velocity data were modified using the intention estimation scheme; A, acceleration; PVI, position-velocity interaction; T, target; SH, spiking history of population; Self history, using past spiking of neuron to predict its future spiking; Others history, using past spiking of other neurons in population to predict future spiking; Population history, using past spiking of all neurons in population to predict future spiking. For details, see text. Pooled: combining data from two monkeys. Top 10 percentile units: using only units with mean spike height (peak to trough) in the top 10 percentile of all units in the session*.

We first plotted the distribution of encoding model prediction accuracy (in CC), among all units in all sessions, as a histogram in Figure 4A. In this figure, the dashed vertical lines represent the mean correlation values for each model. We see that the UKF2 model predicted spike counts more accurately for many unit instances (which include repeated observations of the same units over different sessions), with substantially higher mean prediction accuracy (UKF2: 0.210, p-v Kalman: 0.098, UKF1: 0.138). These values are substantially lower than decoding accuracy values, which is not surprising given our limited understanding of what individual neurons are doing and the intrinsic noise in spiking. The fact that we can achieve higher decoding accuracies despite this is because the decoding algorithm is aggregating information from hundreds of units.

We wanted to know how each new enhancement of the UKF2 model contributed to the improvement in encoding accuracy. In addition to the four improvements, we also tested intention estimation (Gilja et al., 2012; Fan et al., 2014), applying it to both training and testing data, to see if this improves encoding accuracy for the Kalman filter's position-velocity linear model. We plot the resulting mean correlation coefficient and SNR in Figure 4B. We see that adding spiking history to the UKF1 model resulted in the largest increase in accuracy. Adding the other features resulted in smaller increases in accuracy. Intention estimation did not improve the mean encoding accuracy of the position-velocity linear model.

We wanted to know if these improvements were significant. However, the prediction accuracy of different units is not independent in this analysis since units from different sessions may be the same neuron. Thus, we chose one session from each monkey to perform significance testing. We chose a relatively long center-out session for each monkey with a large number of units sorted. We show the mean prediction accuracy for one session from each monkey in Figure 4C. As measured by correlation, ANOVA found a main effect on decoding method [monkey B: *F*_(10, 410)_ = 716.58, *p* < 10^−10^, monkey M: *F*_(10, 218)_ = 96.68, *p* < 10^−10^]. *Post-hoc* comparisons showed that the mean prediction accuracy of the UKF2 model was significantly higher than that of the position-velocity Kalman and UKF1 models for both monkeys (all corrected *p* < 10^−6^). The differences in mean prediction accuracies were all significant (all corrected *p* < 10^−6^) between UKF1 and UKF1 augmented with each enhancement, as well as between UKF2 and UKF1 augmented with each enhancement. The UKF1 model also had significantly higher (corrected *p* < 10^−6^) mean prediction accuracy than the position-velocity linear model of the Kalman filter decoder. Comparing the contribution of different enhancements when added to UKF1, all pair-wise tests were significant (all corrected *p* < 10^−6^), except UKF1+A vs. UKF1+PVI for monkey M (corrected *p* = 0.25). For both monkeys, the linear position-velocity model with intention estimation had lower mean correlation than without intention estimation (corrected *p* ≤ 1.7 × 10^−5^).

We wondered whether the trends in encoding model prediction accuracies would be the same when we only consider units which are more likely to be single units. Thus, we looked at units whose spike height (peak to trough) was in the top 10 percentile of units in their respective sessions. These are the units most likely to be well-isolated single units, and the encoding model prediction accuracies for them were higher under all models. We plot the mean prediction accuracy for top 10 percentile spike height units in Figure 4D. ANOVA found a main effect on decoding method [monkey B: *F*_(10, 40)_ = 53.96, *p* < 10^−10^, monkey M: *F*_(10, 20)_ = 4.96, *p* < 10^−10^]. Some *post-hoc* comparisons between features were no longer significant, particularly for monkey M, since the amount of data was less (monkey B: *n* = 41, monkey M: *n* = 21), but the trends remained the same.

We were curious how much spiking history alone could predict firing rates. Thus, we compared three simple encoding models that did not use any kinematics or target position, only firing rate history. The *self history* model uses one bin of firing rate history of neuron *i* to predict neuron *i*'s instantaneous firing rate:

(22) $$\begin{array}{l} {fr}_{i,\;t}^{\text{self history}}\approx c\cdot {fr}_{i,\;t\;-\;1}^{\text{actual}}, \end{array}$$

where *c* is a fitted coefficient. The *others history* model uses one bin of the firing rate history of the entire population, except the neuron we are trying to model:

(23) $$\begin{array}{rcl} {fr}_{i,\;t}^{\text{others history}} & \approx & c\cdot {fr}_{1,\;t\;-\;1}^{\text{actual}}+\cdots +c\cdot {fr}_{i\;-\;1,\;t\;-\;1}^{\text{actual}}+c\cdot {fr}_{i\;+\;1,\;t\;-\;1}^{actual}\\ +\cdots +c\cdot {fr}_{n,\;t\;-\;1}^{\text{actual}}, \end{array}$$

where *c*s are *n*-1 different fitted coefficients, and *n* is the size of the population. The *population history* model uses one bin of the entire population, including the neuron we are trying to model:

(24) $$\begin{array}{l} {fr}_{i,\;t}^{\text{population history}}\approx c\cdot {fr}_{1,\;t\;-\;1}^{\text{actual}}+\cdots +c\cdot {fr}_{i,\;t\;-\;1}^{\text{actual}}+\cdots +c\cdot {fr}_{n,\;t\;-\;1}^{\text{actual}}, \end{array}$$

where *c*s are *n* different fitted coefficients. The mean model prediction correlations are shown in Figures 4C,D in the right-most bars.

When considering all units (Figure 4C), self history had the lowest accuracy. Others history was substantially higher, and population history was only a small amount higher than others history. Pair-wise differences between the three models were all significant (all corrected *p* ≤ 2.0 × 10^−6^). Notably, for monkey B, all three models were significantly more accurate than the position-velocity model with and without intention estimation (all corrected *p* < 10^−6^), and the others history and population history models were significantly more accurate than the UKF1 model (all corrected *p* < 10^−6^). For monkey M, the others history and population history models were significantly more accurate than the position-velocity model with and without intention estimation (all corrected *p* < 10^−6^) and UKF1 (*p* < 0.00076). The UKF2 and UKF1+SH models were significantly more accurate than all three history models for both monkeys (all corrected *p* < 10^−6^), which is expected since they include the history models.

When considering top 10 percentile spike height units (Figure 4D), the trends are similar, but fewer comparisons were significant due to less data. Population history was significantly more accurate than the self history model for both monkeys (all corrected *p* ≤ 0.0033). For monkey B, all three history models were significantly more accurate than the position-velocity model with and without intention estimation and the UKF1 model (all corrected *p* ≤ 0.0013). For monkey M, differences between the three history models vs. the position-velocity model (with and without intention estimation) and vs. the UKF1 model were not significant. The UKF2 and UKF1+SH models were significantly more accurate than all three history models for both monkeys (all corrected *p* ≤ 0.00086), as expected.

**Figure 5:**
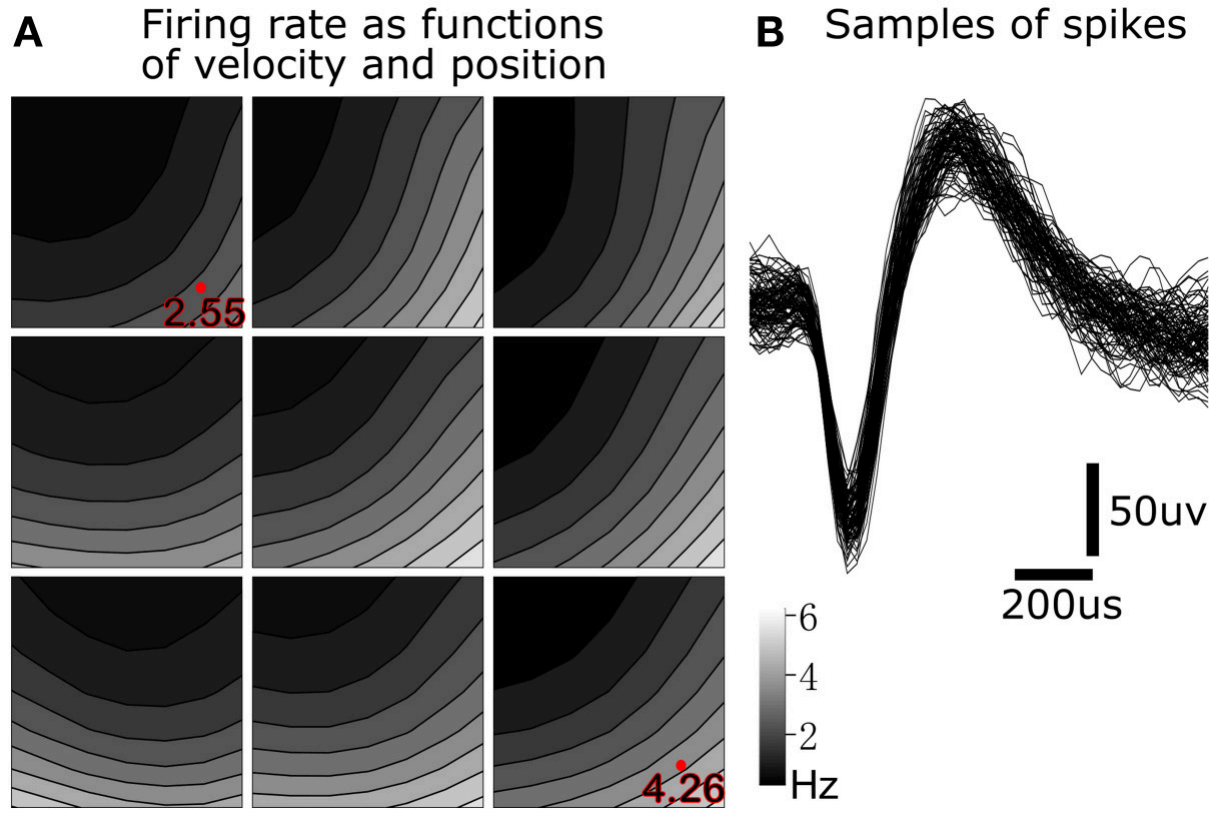
**Position-velocity interaction in the encoding of a motor cortical single unit. (A)** Illustration of position-velocity interaction tuning. Shading indicates firing rate. Each sub-panel depicts velocity tuning when the cursor was in a portion of the position work space, with the sub-panel's position corresponding to the cursor position. Location within each sub-panel corresponds to the 2D cursor velocity, with zero velocity in the center. See text for details. **(B)** Example spike waveforms from this single unit.

To illustrate tuning to position-velocity interactions, we depict an example single unit from monkey B with relatively strong position-velocity interaction in Figure 5. Figure 5B shows representative spike shapes from this single-unit. Figure 5A consists of nine panels, where each panel shows tuning in a different portion of the work space. For example, the lower right panel shows velocity tuning when the cursor is near the lower right (hand is near the right and posterior) portion of the work space. Within each panel, the axes represent cursor velocity, with the center of the panel representing zero velocity. Firing rate is indicated by the shading. For example, the shading in the lower right of a panel is the firing rate of the single unit when the hand is moving toward the right and posterior. The visualization was created by performing Gaussian kernel smoothing on a 7 by 7 grid (per panel). All kinematic variables were normalized to be unit-variance and the smoothing kernel width was 3.

We can see differences in the velocity tuning at different positions in the work space. For example, the firing rate was higher for lower-right velocities in the lower-right position (4.26 Hz at the red dot) than in the upper-left position (2.55 Hz at the red dot). This suggests a multiplicative interaction between position and velocity. This figure illustrates why, for 349 out of 411 (monkey B) and 153 out of 219 (monkey M) units, UKF1+PVI predicted spiking rates better than UKF1, and why PVI is a tuning phenomenon which next-generation neural encoding models should probably take into account.

### Closed-loop neural control experiments

**Figure 6:**
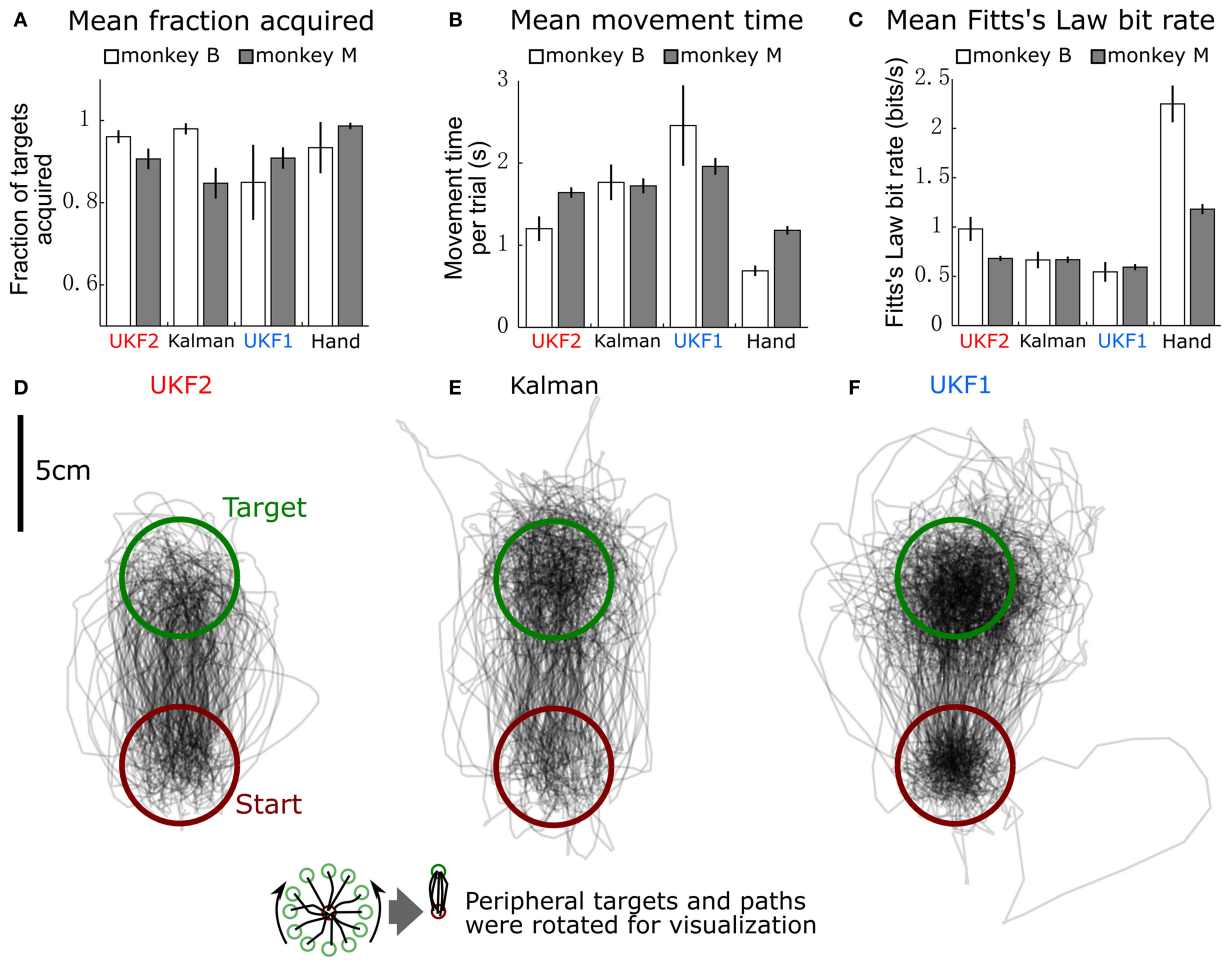
**Comparison of decoders during closed-loop neural control of cursor. (A)** Mean ± SEM of fraction of targets acquired. “Hand” indicates performance when monkey controlled the cursor using its hand via the joystick. **(B)** Mean ± SEM of movement time per peripheral target. **(C)** Mean ± SEM of Fitts's Law bit rate. **(D)** Example movement trajectories generated under UKF2 control. Peripheral targets and paths have been rotated so that all peripheral targets align. **(E)** Trajectories under Kalman control. **(F)** Trajectories under UKF1 control.

We compared the ability of monkeys to complete a center-out task using the decoders in closed-loop neural control. We recorded 7 sessions with monkey B, 153–184 days post-implant, and 30 sessions with monkey M, 15–70 days post-implant. Each session was recorded on a separate day and contains one 10-min block of each condition (UKF2, KF, UKF1), and the last 5 min of each 10-min block were analyzed for performance. We recorded fewer sessions with monkey B because the difference between decoders was larger, and monkey B was tasked with other experiments. The results of closed-loop comparisons are summarized in Table 3 and graphed in Figure 6.

**Table 3:** **Comparison of decoders during closed-loop neural control of cursor**.

**Mean ± SEM**	**Fraction of targets acquired, monkey B**	**Fraction of targets acquired, monkey M**	**Movement time (s), monkey B**	**Movement time (s), monkey M**	**Movement time (s), pooled**	**Fitts's Law bit rate (bits/s), monkey B**	**Fitts's Law bit rate (bits/s), monkey M**	**Fitts's Law bit rate (bits/s), pooled**
UKF2	0.961 ± 0.016	0.906 ± 0.025	1.201 ± 0.150	1.640 ± 0.065	1.557 ± 0.065	0.980 ± 0.122	0.682 ± 0.026	0.738 ± 0.036
FIT Kalman filter	0.980 ± 0.014	0.847 ± 0.037	1.766 ± 0.217	1.722 ± 0.092	1.730 ± 0.083	0.666 ± 0.084	0.668 ± 0.032	0.668 ± 0.030
UKF1	0.850 ± 0.091	0.909 ± 0.026	2.456 ± 0.489	1.959 ± 0.103	2.053 ± 0.124	0.545 ± 0.100	0.593 ± 0.032	0.584 ± 0.031
Hand control	0.934 ± 0.062	0.987 ± 0.008	0.689 ± 0.065	1.182 ± 0.050	1.089 ± 0.053	2.248 ± 0.186	1.178 ± 0.050	1.381 ± 0.087

*Pooled, combining data from two monkeys*.

We calculated the mean (across sessions) of the fraction of targets acquired (Figure 6A), time to move to a peripheral target (Figure 6B), and Fitts's Law bit rate (Figure 6C) for each decoder, as well as hand control of the cursor via joystick.

The fractions of targets acquired were not significantly different between conditions for monkey B [ANOVA *F*_(3, 6)_ = 1.13, *p* = 0.36]. For monkey M, ANOVA found a main effect on mode of control [*F*_(3, 29)_ = 5.4474, *p* = 0.0018], and *post-hoc* tests showed that fractions of targets acquired for the decoders were not significantly different, but all decoders had significantly lower fractions than hand control (all corrected *p* < 0.034). The high variance in fraction of targets acquired by monkey B (session MS = 0.02718, compared to control condition MS = 0.02309, while other closed-loop control metrics had control condition MS which were 4–20 times larger than session MS) was due to poor parameter fits in some experimental sessions leading to relatively poorer control, which led to monkey non-participation during the evaluation period, as the monkey had grown used to very good neural control.

In terms of time to move to a peripheral target, for monkey B, ANOVA found a main effect on mode of control [*F*_(3, 6)_ = 9.9, *p* = 0.0004]. *Post-hoc* tests showed that UKF2 times were significantly shorter than UKF1 times (corrected *p* = 0.049), other decoder comparisons were not significant. Hand control had significantly shorter times than every decoder (all corrected *p* < 0.040). For monkey M, ANOVA found a main effect on mode of control [*F*_(3, 29)_ = 36.8127, *p* = 1.87 × 10^−15^], and *post-hoc* tests showed that UKF2 and FIT Kalman filter times were significantly shorter than UKF1 times (corrected *p* = 0.0012, *p* = 0.020, respectively), the UKF2 and FIT Kalman comparison was not significant. Hand control had significantly shorter times than every decoder (all corrected *p* <2 × 10^−6^).

In terms of Fitts's Law bit rates, for monkey B, ANOVA found a main effect on mode of control [*F*_(3, 6)_ = 90.09, *p* < 10^−10^]. *Post-hoc* tests showed that UKF2 bit rates were significantly higher than UKF1 bit rates (corrected *p* = 0.00061), and other decoder comparisons were not significant. Hand control had significantly higher bit rate than every decoder (all corrected *p* < 0.00057). For monkey M, ANOVA found a main effect on mode of control [*F*_(3, 29)_ = 155.2043, *p* = 8.2 × 10^−35^]. *Post-hoc* tests showed that UKF2 and FIT Kalman filter bit rates were significantly higher than UKF1 bit rates (corrected *p* = 0.0030, *p* = 0.012, respectively). UKF2 bit rates and FIT Kalman bit rates were not significantly different, and hand control had significantly higher bit rate than every decoder (all corrected *p* < 10^−6^).

When we pooled the data between the two monkeys, the mean movement times were significantly different among the three decoders and hand control [ANOVA *F*_(3, 36)_ = 34.94, *p* = 7.3 × 10^−16^, *post-hoc* UKF2 < FIT Kalman, corrected *p* = 0.045; UKF2 < UKF1, corrected *p* = 0.00024; FIT Kalman < UKF1, corrected *p* = 0.013]. Mean Fitts's law bit rates were also significantly different among the three decoders and hand control [ANOVA *F*_(3, 36)_ = 80.19, *p* = 2.3 × 10^−27^, *post-hoc* UKF2 > FIT Kalman corrected *p* = 0.046; UKF2 > UKF1 corrected *p* = 0.00006; FIT Kalman > UKF1 corrected *p* = 0.0039]. The hand movement times (all corrected *p* < 10^−6^), and bit rates (all corrected *p* < 10^−6^) were significantly better than that for every decoder. For fraction of targets acquired, there was a main effect on mode of control [ANOVA *F*_(3, 36)_ = 3.81, *p* = 0.012], but *post-hoc* testing did not find significant differences among the decoders. The hand control fraction correct was better than that for the FIT Kalman (corrected *p* = 0.036) and UKF1 (corrected *p* = 0.040).

Figures 6D–F show all center to peripheral movement trajectories generated during UKF2 control, FIT Kalman filter control, and UKF1 control, respectively, for one session from monkey B. For clarity of visualization, the trajectories have been rotated so that the peripheral target locations are aligned at the top. Thus, all trajectories start from the lower, red circle and end in the upper, green circle. We can see that the trajectories generated during UKF2 control start movement in the wrong direction and overshoot the target the least among the three decoders. From the movement time and bit rate comparisons and the trajectory illustrations, we can see that UKF2 allows the monkey to perform center-out movements more quickly and accurately than the UKF1 and comparably to the FIT Kalman filter.

One may ask why the plot of trajectories for UKF1 (Figure 6F) looks darker within the targets. This is because the decoded cursor positions are more “jumpy” during the hold period. This is due to the use of position as the signal to control the cursor, and noisy neural activity causes the estimated position to jump. We conjecture that the position-velocity mixing scheme (Homer M. et al., 2013) and our probabilistic velocity thresholding refinement solve this problem for the UKF2.

## Discussion

The UKF2 reconstructed kinematics offline more accurately than the position-velocity Kalman filter and UKF1. Examining why the UKF2 performed better, analysis of the encoding model found that the UKF2's encoding model made more accurate predictions of neural activity. In closed-loop neural control experiments, the UKF2 allowed better task performance than the UKF1, but comparisons with the FIT Kalman filter were not significant on a per-monkey basis, though they were significant when data from two monkeys were combined.

The differences between monkeys were quite large. Monkey B was more proficient at the center-out task under hand control, with lower movement times and higher bit rate. Offline reconstructions were generally more accurate with monkey B. The differences between decoders in closed-loop control were larger for monkey B. A particularly pronounced area of disparity was encoding model predictions. Monkey B and monkey M had similar encoding model prediction accuracy for the Kalman filter, but quite different accuracy for the UKF1 and UKF2. For monkey B, adding spiking history to UKF1 resulted in the largest improvement, with target tuning also large. For monkey M, improvements from these two features were substantially smaller. The trends suggest that spiking history is less beneficial for modeling the activity of monkey M's units, and even harmful for decoding accuracy, which hints at some qualitative differences in the populations recorded from these two monkeys. Overall, the differences between monkeys may be due to: electrode length (B: 1.0 mm, M: 1.5 mm), age (B: 6, M: 4), amount of practice with the center-out task (B: 4 months, M: 2 months), and spike sorting performed by different experimenters.

During the design of the UKF2 algorithm, we used pursuit task data from another monkey, which we cannot publish here. We froze the design of our algorithm, as much as possible, before starting closed-loop experiments and data collection for reconstructions. Thus, we included refinements such as PVI and SH which were not universally beneficial.

Our results with CC and SNR are, in some cases, quite different in absolute terms. This is understandable since they are very different measures, with SNR including a logarithm to avoid saturation.

All decoders we tested, including the FIT Kalman filter, had significantly worse performance, in terms of movement time and Fitts's Law bit rate, than hand control via the joystick. These differences were large, for example, hand control had 1.9 times the bit rate of UKF2 and 2.1 times the bit rate of the FIT Kalman filter. This indicates there is still much room for improvement in decoding and signal acquisition methodology.

In terms of the contributions of the different refinements, our closed-loop results are limited in granularity; future work may investigate the individual contributions in closed-loop, which would greatly assist practitioners in optimizing their decoder design.

### Acceleration

Acceleration of the hand has been decoded in past studies (Ashe and Georgopoulos, 1994; Gao et al., 2003), and force has long been known to contribute to encoding (Evarts, 1968 and many others). In our previous work, we considered using acceleration as a feature in the encoding model, but did not detect a substantial benefit. After UKF1's publication, we continued to look for ways to improve the neural encoding model using new kinematic features. Our recent analysis included temporal smoothing (sliding window moving average) of kinematics before parameter fitting for the encoding model (see Supplementary Materials). Doing this, the quickly-changing acceleration signal is smoothed out, and neural encoding strength for acceleration is increased, resulting in worthwhile decoding accuracy increases. Similar to position and velocity, upon visualization of acceleration tuning, we found spatial patterns consistent with the existence of a relationship between the acceleration magnitude (in any direction) and the firing rate of many neurons. Thus, we have also included the magnitude of the acceleration vector as a novel feature of the UKF2 encoding model. Even though we do not use the acceleration in the filter state to directly control the cursor, the acceleration interacts with the other variables in the state space via the state transition model (movement model).

### Position-velocity interaction

In our quest for a better encoding model, we visualized neural tuning patterns in various ways (for example, Figure 5A). We found that the encoding of velocity changes with the position of the hand in a systematic way. Thus, we surmised that there is an interaction between position and velocity tuning. Differences in preferred directions at different limb postures have previously been found (Caminiti et al., 1990; Sergio and Kalaska, 2003), and gain-field encoding has been suggested for limb position and velocity (Hwang et al., 2003). To capture this interaction, we added a multiplicative feature to our encoding model which is a simple multiplication of the position and velocity for each dimension separately. This encoding model refinement was used both in offline and closed-loop decoding. We found that by adding this novel encoding feature, significantly more accurate predictions of firing rate could be achieved (Figure 4C).

However, in terms of offline reconstructions, using position-velocity interaction was actually detrimental when considering both center-out and pursuit tasks, and slightly helpful (though not significant, since *n* = 6) when considering the pursuit task only. In the center-out task, position (with respect to center of workspace) and velocity are either very correlated (outward movement), very anti-correlated (inward movement), or independent (hold), with few instances of other relationships. This is not true for the pursuit task, which samples the possible space of position and velocity values much more thoroughly. We believe this difference accounts for the results we found. For neural control of a prosthetic, where movements throughout the space of possible position and velocity values need to be supported, the position-velocity interaction term will likely help.

### Target

Inspired by studies which included the target of reaches in the trajectory decoding process (Shanechi et al., 2012; Shanechi M. et al., 2013; Shanechi M. M. et al., 2013; Shanechi and Carmena, 2013), we investigated adding information about the target into the encoding model. In preliminary analysis we found that some neurons show significant encoding of target position, which has been found in the past (Fu et al., 1995). Though using the neurons we recorded to decode target position alone provides very noisy results, the rough information that can be decoded is still valuable. We set the target position to weakly attract the cursor during closed-loop control. This, in effect, gives a small assistance to the cursor decoding by using the rough estimate of the target location. This is somewhat similar to the mechanism that Shanechi and Carmena (2013) used, where the target position is used in an optimal feedback controller, which can be understood as biasing decoded movement toward the target.

In addition to target position in Cartesian coordinates, our preliminary analysis showed that there was significant encoding of the distance between target position and cursor position, which is similar to the reach distance found by Fu et al. (1995). Thus, we included this novel feature in our encoding model as well.

### Spiking history

In multiple previous studies (Paninski et al., 2004b; Truccolo et al., 2005; Lawhern et al., 2010; Saleh et al., 2010, 2012; Truccolo et al., 2010; Park et al., 2014; Xu et al., 2014) the past spiking of a neuron as well as the past spiking of other neurons in the population have been used to better model the probability of spiking in a point process framework, sometimes leading to very accurate models (Truccolo et al., 2010). The past neural activity of all neurons in the population may capture correlations in firing due to functional connectivity or common inputs. Another advantage of this modeling approach is the ability to indirectly capture tuning to latent neural states. One disadvantage of this approach is the large number of additional parameters that must be fitted, with the accompanying increase in over-fitting risk.

We wanted to include spiking history in our encoding model to capture these benefits. However, instead of using complex temporal features as in the point process studies, we use a comparatively simple idea: we include only the spike counts in the previous bin for the entire population. In preliminary analysis we investigated using more than one previous bin and found the benefit to be small in comparison to the additional cost in number of parameters that needed to be fit.

During closed-loop decoding, the spike count of the previous time bin is available, and is directly provided to the neural encoding model as point values without uncertainty. In other words, unlike the filter state variables in the encoding model, the previous spike counts are not decoded; their benefit comes from improving the fit of the encoding model. This is similar to the role of the position state variables in the ReFIT Kalman filter. By including spiking history, we aim to remove more systematic variation (e.g., from autocorrelation) from the encoding model's residual, making the remaining residual closer to white noise, which better fits the theoretical assumptions of the Kalman filter framework. However, it is still beneficial to include improved kinematic features in the encoding model: these provide the “conduits” through which information flows from neural activity to kinematic variables during the operation of the unscented Kalman filter.

Our results show that adding spiking history improved encoding model predictions substantially. However, offline reconstruction accuracy only improved when adding spiking history for monkey B, with possible reasons discussed above.

### Mixing position and velocity outputs

In our previous work with the unscented Kalman filter, we used the decoder's position output to directly drive the cursor. As the position is decoded from noisy firing rates, we observed that the decoded position jumped around in a small area at high frequency. One benefit of the position-as-feedback enhancement of the ReFIT Kalman filter is that by controlling position only through decoded velocity, this “jumpy” cursor phenomenon is avoided.

We believe that while some neurons in the motor cortex do encode for the current position of the limb in a feedback or mental representation sense, there also exist neurons which encode for the desired position in the immediate future (as opposed a distant future time, i.e., the ultimate target of a reach). If we do not make use of encoded desired position, we may be losing information potentially helpful for controlling a neuroprosthetic, particularly because UKF2's encoding model includes position-velocity interaction terms.

Thus, we chose to retain position as a directly decoded variable in the state space. However, in an effort to avoid the “jumpy” cursor of the UKF1, we adopted the method for mixing position and velocity proposed by Homer M. et al. (2013). In this scheme, if the decoded velocity is zero, the position decode cannot change the cursor. This mechanism assists the user in stopping the cursor, as well as reduces the jumpiness of the cursor. By partially controlling the cursor using the position output, we also stabilize the cursor, preventing velocity decode errors from accumulating, which was a problem we discovered in preliminary experiments when combining the position-velocity interaction enhancement and the position-as-feedback refinement of the ReFIT Kalman filter.

### Movement thresholding

The ability to stop and hold the cursor (or prosthetic limb) is important for various tasks. Several studies have examined how to stop the cursor more accurately. Golub et al. (2014) used a refinement of the transition model which allows the user to perform a “hockey stop,” that is, the user changes the movement direction quickly to slow the cursor to a stop. Another approach (Velliste et al., 2014) decoded a speed term, separately from the Cartesian velocity coordinates. This speed term is used to scale the position and velocity uncertainties in the transition model, effectively acting as a gate for movements. In the mixture method of Homer M. et al. (2013), decoded velocity also acted like a gate for the influence of decoded position on the cursor. Another related decoding engineering feature is the detection of idle states—when the user is not actively using the neuroprosthetic. Aggarwal et al. (2013) and Velliste et al. (2014) detected states using a linear discriminant analysis classifier, separate from the movement decoder. When an idle or hold state was detected, the decoder output is ignored and movement was set to zero. Recently, Sachs et al. (2016) detected posture vs. movement states using linear discriminant analysis and used Wiener filters with different coefficients during each.

To improve the user's ability to stop the cursor within the target during closed-loop control, we added our own mechanism to detect movement intention, a probabilistic threshold for movement which is computed using the uncertainty output of the unscented Kalman filter. Using a probabilistic threshold means we can set the threshold in terms of a false positive rate. This probabilistic threshold is similar to a significance test; the null hypothesis is that the user wants to remain still. We check if there is enough evidence to reject the null hypothesis under the specified false positive rate. This framework, while more complex than a simple threshold on the decoded velocity, allows one threshold to work under different amounts of uncertainty in the decoded output, e.g., for both fast and imprecise (more uncertain) movements and slower and more precise (more certain) movements.

In this study, we set the desired false positive rate by hand. Larger false positive rates mean the cursor is rarely stopped through this mechanism, and holding inside a target may be more difficult if control is poor. Smaller false positive rates may make the cursor too difficult to move. Future decoders with multiple modes of operation may find it advantageous to use a lower false positive rate for certain modes where unwanted movements are dangerous or highly undesirable, e.g., when the user is asleep. While the position and velocity mixing method (Homer M. et al., 2013) also helps stop the cursor, it is dependent on accurate decoding of velocity. Our probabilistic threshold complements this method by verifying that the velocity is not non-zero due to mere noise.

### Use of future predictions

Motor cortex neurons encode for movements that occur up to a few hundreds of milliseconds later (Ashe and Georgopoulos, 1994; Schwartz et al., 2004; Paninski et al., 2004a; Wu et al., 2006; Wang et al., 2007; Wang and Principe, 2010), making decoding of intentions at *t* + 100 ms to *t* + 300 ms possible given neural activity at time *t*. These “future predictions” are merely a reflection of the built-in delays in the natural motor system. Most previous work used the decoded kinematics for time *t* as the output at time *t*, in effect mimicking the delay of the natural motor system. In the UKF1, even though future intended movements are decoded, we did not choose to use future predictions to control the cursor.

Cunningham et al. (2011) found that reducing the bin width during closed-loop neural control with feedback improves performance, and Willett et al. (2013) found that using predictions of future intentions can compensate for delays in the BMI system. Inspired by these studies, we wondered whether using future predictions could improve neural control. Our temporal offset was 100 ms in size, i.e., we use the decoded kinematics for *t* + 100 at time *t*, which is reasonable considering the 75–100 ms average offset found in prior work (Ashe and Georgopoulos, 1994; Paninski et al., 2004a; Schwartz et al., 2004).

One may ask why include the other taps in the filter state, if they are not used to control the cursor. The answer is that they help model the firing rate of neurons, which may encode for movements in a temporally persistent manner or specifically encode for movements in the past. Similar to adding spiking history to the encoding model or the position-as-feedback enhancement, the other taps do not directly affect the decoder output, but may improve decoder accuracy by explicitly modeling what would otherwise be thrown in to the catch-all error term.

### Related work

Reviews of research in decoding for BMIs can be found elsewhere (Homer M. L. et al., 2013; Andersen et al., 2014; Baranauskas, 2014; Bensmaia and Miller, 2014; Kao et al., 2014; Li, 2014). Here we discuss the decoders compared in the present study.

The improved unscented Kalman filter decoder proposed in this study is a development of our previous unscented Kalman filter decoder (Li et al., 2009). That filter, which we refer to here as UKF1, used an encoding model with non-linear dependence on kinematic variables which modeled tuning to the speed or velocity magnitude of movements. The UKF1 modeled tuning at multiple temporal offsets, using an n-th order hidden Markov model framework where n taps of kinematics (*n* = 10 was tested) are held in the state space. Encoding studies by Paninski et al. (2004a,b), Hatsopoulos et al. (2007), Hatsopoulos and Amit (2012) and Saleh et al. (2010) found tuning to position and velocity trajectories, called movement fragments or pathlets. The n-th order framework makes the encoding model of the UKF1 flexible enough to capture such tuning. Even though including taps of position also indirectly includes velocity, explicitly including taps of velocity reduces the amount of non-linearity needed in the neural encoding model, which helps improve the approximation accuracy of the UKF. On the basis of UKF1, we expand the neural encoding model and add decoder engineering improvements developed by ourselves and other groups to make the UKF2.

The ReFIT Kalman filter (Gilja et al., 2012) has demonstrated high communications bit rate by using two advances in decoder engineering. In closed-loop experiments, we compared the UKF2 with the FIT Kalman filter (Fan et al., 2014), which is similar to the ReFIT Kalman filter in using position-as-feedback and intention estimation, but does not have the online re-training component. The bin size in this study, 50 ms, was the same as the Gilja et al. study. Our Fitts's Law bit rate values for the FIT Kalman filter are lower than those reported by Gilja et al. for the ReFIT Kalman filter, likely due to a combination of factors. First, online re-training separates the FIT and ReFIT Kalman filters. In terms of experimental setup, Gilja et al. used video tracking of natural reaching movements, whereas we used a joystick during hand control of the cursor. The use of an unnatural joystick made our task more difficult: the mean movement time during hand control in our task was approximately double those reported by Gilja et al. we used a joystick due to the limitations of our experimental platform and to compare with our previous work (Li et al., 2009). While using the same Fitts's law bit rate measure, our task used circular targets, which have a smaller acceptance area for the same width compared to the square targets of Gilja et al. We used circular targets because they are more natural in terms of determining whether the cursor is within the target by using a distance criterion. We also spike sorted and did not include unsorted or “hash” threshold crossings, whereas Gilja et al. used threshold crossing counts.

### Latent neural state

Models proposed by many previous studies have modeled latent neural states explicitly (Brockwell et al., 2007; Kulkarni and Paninski, 2007; Wu et al., 2009; Lawhern et al., 2010; Macke et al., 2011; Petreska et al., 2011; Aghagolzadeh and Truccolo, 2014, 2016; Deng et al., 2015; Kao et al., 2015; Lakshmanan et al., 2015). In Aghagolzadeh and Truccolo (2014) and Kao et al. (2015), the latent neural state comprises the entirety of the Kalman filter state, and kinematics are decoded from this latent neural state after it is decoded from the spike counts. When modeling latent states like this, some form of unsupervised learning is required to fit the observation model of the filter. The typical approach is Expectation-Maximization applied to linear dynamical systems (Shumway and Stoffer, 1982).

An alternative approach is to implicitly model latent states by adding spiking history to the observation model. By adding spiking history, one may (partially) capture latent shared variables if they have temporal autocorrelation. In other words, if a unobserved common input of many neurons is changing slowly, by using the past neural activity, which partially encodes this hidden input, to predict the current neural activity, one is including this hidden common input in the encoding model by proxy.

Two engineering advantages of explicitly modeling the latent variable using additional state variables are: (1) lower dimensionality; (2) ability to impose prior assumptions on the model structure, such as in the transition model or observation model (Aghagolzadeh and Truccolo, 2016). Additionally, investigating these latent states may yield neuroscientific insights. However, it is not obvious this approach is always better from a decoding point of view, since the unsupervised learning of latent variables cannot be checked against a gold standard, and, in practice, it is vulnerable to local optima. Some of the autocorrelation or cross-correlation captured by spiking history may not be due to low-dimensional latent variables, but are due to biophysics and actual neuronal connectivity. The effect of these phenomena may not be easily captured by low dimensional latent states.

## Conclusion

We have shown in offline analysis and closed-loop experiments with two Rhesus monkeys that our encoding model features and decoder engineering refinements improve encoding and decoding accuracy. Some of the enhancements used in this work, particularly the probabilistic velocity thresholding and the inclusion of hand acceleration and target position (without the non-linear terms) are compatible with the standard Kalman filter, are fairly easy to implement, and are likely to bring the largest benefits. We hope that these enhancements will be utilized by others, just as we have improved our decoder using innovations published by others.

## Author contributions

SL and ZL designed the algorithm. SL and JL collected the data. SL, JL, and ZL analyzed the results. ZL drafted the manuscript. SL, JL, and ZL revised the manuscript.

### Conflict of interest statement

The authors declare that the research was conducted in the absence of any commercial or financial relationships that could be construed as a potential conflict of interest.
