# Supplementary material for: An Improved Unscented Kalman Filter Based Decoder for Cortical Brain-Machine Interfaces
Source: Front Neurosci. 2016 Dec 22;10:587. doi: 10.3389/fnins.2016.00587 (PMC5177654; doi:10.3389/fnins.2016.00587)
Supplement: Supplementary file 1 [file DataSheet1.pdf]

Supplementary Material

An improved unscented Kalman filter based decoder for cortical brain-machine interfaces

Simin Li[1,2], Jie Li[1,2], Zheng Li[1,2]*

*** Correspondence:** Zheng Li: lz@bnu.edu.cn

1 Kalman Filter and Unscented Kalman Filter Overview

Here, we outline the Kalman filter and unscented Kalman filter decoder algorithms. For details on the Kalman filter, see Welch and Bishop (2006). For further details on the unscented Kalman filter decoder, see Li et al (2009). For details on the general unscented Kalman filter, see Julier et al (1995, 2000, 2002).

In the Kalman filter framework, the goal is to estimate or track some variables of interest which cannot be directly observed. These variables can be indirectly observed through some observations, which have a known relationship with the variables of interest. In the version presented here, time is discretized uniformly and the function describing the temporal evolution of the variables of interest is known. Both the observations and the temporal evolution are contaminated with noise, and the noise is assumed to have known covariance (across dimensions) and assumed to be independent across time.

In neural decoding for cursor or prosthesis control, the variables of interest are kinematics and potentially other variables to decode. The observations are binned spike counts of the recorded population or some other quantification of neural activity.

Let the true state (kinematics) at time *t* be the m-dimensional column vector $\bar{s}_t$. Let the observations (spike counts) at time *t* be the n-dimensional column vector $\bar{y}_t$. Then we assume we have knowledge of how the states relate to the observations:

$$\bar{y}_t = h(\bar{s}_t) + \bar{\epsilon}_{\mathrm{h}}, \qquad (1)$$

where $h()$ is a function called the observation model, and $\bar{\epsilon}_{\mathrm{h}}$ is zero mean independent (across time) random noise with known covariance matrix *R*. In the standard way of applying the Kalman filter to neural decoding, the observation model $h()$ is the neural encoding model which generates predictions of firing rate given kinematics and other variables of interest. For the Kalman filter, the function $h()$ is a linear function and is written as a multiplication with a weight matrix *H*. For the unscented Kalman filter, $h()$ can be non-linear, though non-linear functions which change rapidly carry a higher approximation penalty. We can fit the neural encoding model, that is, fit the weights in the matrix *H* or the parameters of the function $h()$, by using training data that consists of recordings of neural activity and concurrent kinematics.

We also assume we know how the state evolves with time:

$$\bar{s}_{t+1} = f(\bar{s}_t) + \bar{\epsilon}_{\mathrm{f}}, \tag{2}$$

where $f()$ is a function called the transition model, and $\bar{\epsilon}_{\mathrm{f}}$ is zero mean independent (across time) random noise with known covariance matrix Q. The function $f()$ is again a linear function for Kalman filtering, and it is written as a multiplication with a weight matrix F. For the UKF1 and UKF2, $f()$ is also a linear function, as we do not focus on sophisticated modeling of kinematics.

The above equations describe the modeling framework for Kalman filtering. During operation, the filter does not have access to the true states, but generates estimates for them. Our estimates have some uncertainty so we represent the uncertainty with a probability distribution, which we parameterize by its mean and covariance. Let us denote the (m x 1) mean vector of the estimate of the state variables at time *t* as $\tilde{s}_t$. Let us denote the (m x m) covariance matrix of the estimate of the state variables at time *t* as P_t.

The equations implementing the Kalman filter, without control inputs, are (Welch and Bishop 2006):

$$\tilde{s}_t^{'} = F\tilde{s}_{t-1}, \tag{3}$$

$$P'_t = FP_{t-1}F^{\top} + Q, \tag{4}$$

$$K_t = P'_tH^{\top}\left(HP'_tH^{\top} + R\right)^{-1}, \tag{5}$$

$$\tilde{s}_t = \tilde{s}_t^{'} + K_t\left(\bar{y}_t - H\tilde{s}_t^{'}\right), \tag{6}$$

$$P_t = (I - K_tH)P'_t, \tag{7}$$

where $\tilde{s}_t^{'}$ (m x 1) and P'_t (m x m) are intermediate variables, K_t (m x n) is the Kalman gain at time *t*, and I is the m-dimensional identity matrix. These equations are executed once per time step (corresponding to one bin). The estimated mean state variables $\tilde{s}_t$ are used as the decoder output at step *t*.

The equations implementing the UKF1 and UKF2 start the same as the Kalman filter:

$$\tilde{s}_t^{'} = F\tilde{s}_{t-1}, \tag{8}$$

$$P'_t = FP_{t-1}F^{\top} + Q. \tag{9}$$

Then, the sigma points $\bar{X}_0 \ldots \bar{X}_{2m}$, a set of deterministic sampling points, are generated from the distribution parameterized by $\tilde{s}_t^{'}$ and P'_t. The purpose of the sigma points is this: a key step in Kalman filtering is the computation of the expected observations (given the current estimate of the state). The filter needs both the mean and covariance matrix of the expected observations. In the

Kalman filter equations above, $H\tilde{s}_t^{'}$ is the mean and $H{P'}_t H^\top + R$ is the covariance. These calculations are closed-form because the observation model is linear. When the observation model is non-linear, there is no general closed-form method for arbitrary non-linear functions. One way to approximate the mean and covariance of the expected observations is via sampling. The unscented transform is a deterministic sampling procedure to do this, and it is the heart of the unscented Kalman filter. This procedure first computes a set of sigma points, then passes these sigma points through the non-linear observation function, and then computes the mean and covariance from the resulting sigma points.

The sigma points are:

$$\bar{X}_0 = \tilde{s}_t^{'}, \tag{10}$$

$$\bar{X}_i = \tilde{s}_t^{'} + \left(\left(\sqrt{(m+1){P'}_t} \right)_{(i)} \right)^\top \qquad i = 1 \ldots m\,, \tag{11}$$

$$\bar{X}_i = \tilde{s}_t^{'} - \left(\left(\sqrt{(m+1){P'}_t} \right)_{(i-m)} \right)^\top \qquad i = m+1 \ldots 2m\,, \tag{12}$$

where $\bar{X}_0 \ldots \bar{X}_{2m}$ are a total of 2m+1 column vectors of length m, the subscript outside the parentheses indicate which row should be taken from the matrix inside the parentheses, and the square root is the matrix square root. The matrix square root is computed using the Cholesky decomposition for robustness.

Next, the sigma points are evaluated through the observation model:

$$\bar{Z}_i = h(\bar{X}_i) \qquad i = 0 \ldots 2m\,, \tag{13}$$

where $\bar{Z}_i$ are 2m+1 column vectors of length n. Evaluating the observation model, or neural encoding model, consists of the following steps. (1) Generate features, including the non-linear terms in the neural encoding model. For the UKF2, augment kinematic features with the spike counts from the previous time step (we include these when referring to features in the following). (2) Subtract from each feature the mean calculated for that feature based on the training data. (3) Divide each feature by the standard deviation calculated for that feature based on the training data. (4) Multiply features by the coefficients fitted from the training data. (5) Add products together to arrive at a (centered and normalized) spike count prediction for each unit. The multiply and add in steps 4 and 5 are implemented using matrix multiplication. The predicted spike count for each unit is an element of the column vector $\bar{Z}_i$. Repeat the above steps for each $\bar{X}_i$ to get the set of 2m+1 vectors $\bar{Z}_i$. Then we find the mean of $\bar{Z}_i$, the covariance of $\bar{Z}_i$, the cross-covariance between $\bar{Z}_i$ and $\bar{X}_i$, and the Kalman gain:

$$\bar{z}_t = \frac{1}{m+1}\bar{Z}_0 + \sum_{i=1}^{2m} \frac{1}{2(m+1)} \bar{Z}_i, \tag{14}$$

$$P_t^{\mathrm{zz}} = \frac{1}{m+1}(\bar{Z}_0 - \bar{z}_t)(\bar{Z}_0 - \bar{z}_t)^\top + \left[\sum_{i=1}^{2m} \frac{1}{2(m+1)}(\bar{Z}_i - \bar{Z}_0)(\bar{Z}_i - \bar{Z}_0)^\top \quad \right] + R, \tag{15}$$

$$P_t^{\mathrm{sz}} = \frac{1}{m+1}(\bar{X}_0 - \tilde{s}_t^{'})(\bar{Z}_0 - \bar{z}_t)^\top + \sum_{i=1}^{2m} \frac{1}{2(m+1)}(\bar{X}_i - \bar{X}_0)(\bar{Z}_i - \bar{Z}_0)^\top \quad , \tag{16}$$

$$K_t^{\mathrm{UKF}} = P_t^{\mathrm{sz}}(P_t^{\mathrm{zz}})^{-1}, \tag{17}$$

where $\bar{z}_t$ (n x 1), P_t^{zz} (n x n), and P_t^{sz} (m x n) are intermediate variables. Finally, the mean and covariance of the state estimate are updated:

$$\tilde{s}_t = \tilde{s}_t^{'} + K_t^{\mathrm{UKF}}(\bar{y}_t - \bar{z}_t), \tag{18}$$

$$P_t = P'_t - K_t^{\mathrm{UKF}}(P_t^{\mathrm{sz}})^\top. \tag{19}$$

The above equations execute one time step of the UKF1 or UKF2.

During offline reconstructions, we use the position and velocity output of the tap with zero temporal offset, i.e. at time *t*, we use px_t, py_t, vx_t, and vy_t. During closed-loop control with the UKF1, we use the position output with zero temporal offset. During closed-loop control with the UKF2, we use the position and velocity output of the tap with 2 temporal offset, i.e. at time *t*, we use px_{t+2}, py_{t+2}, vx_{t+2}, and vy_{t+2}, as input to the position-velocity mixture scheme.

2 Ridge Regression Parameter Selection

The best ridge regression parameter per neuron (unit) was found by search. We set an upper limit (16, arbitrary units) to prevent under-fitting and to limit search time. This search optimized the predicted residual sum of squares (PRESS) statistic. PRESS is the leave-one-out cross-validation error of a linear regression, which can be calculated quickly from the hat matrix (Tarpey 2000):

$$\mathrm{PRESS} = \sum_{i=1}^{N} \left(\frac{e_i}{1 - h_{i,i}} \right)^2, \tag{20}$$

where e_i is the residual for data point i, N is the amount of data, and $h_{i,i}$ is the ith diagonal entry of the hat matrix of the regression. The hat matrix for the ridge regression is $X(X^T X + \alpha^2 I)^{-1} X^T$, where X is the design matrix, α is the ridge regression parameter, and I is the identity matrix. Once the regularization parameter was decided, the entire training data was used for a final fit.

3 Model Fitting Details

In closed-loop experiments, we pre-processed the hand movement training data for the UKF2 by applying temporal smoothing. Smoothing was performed by averaging in a moving window of width 5 (bins) centered on the output bin. Smoothing was applied to the position data before taking

the gradient to compute the velocity data. Smoothing was applied to the velocity data before taking the gradient to compute the acceleration data. For the Kalman filter during closed-loop experiments, instead of smoothing, the training data was adjusted using intention-estimation (Gilja et al. 2012, Fan et al. 2014). For the UKF1, no smoothing or intention estimation was used for closed-loop experiments, as was done in Li et al. (2009).

For offline reconstructions and encoding model predictions, training and testing data hand positions were smoothed (before computing velocity) using a moving window of width 11. This was performed for all decoders.

For all decoders, we transformed both kinematic and spike count data so that they were zero mean and unit variance. The shifting and scaling parameters of this transformation were computed from training data only. Additionally, for the unscented Kalman filters, the non-linear features computed from the state variables were also transformed to be zero mean and unit variance. This was performed as a step after feature generation, both during parameter fitting and filter operation (which used the training data's statistics for shifting and scaling). By making inputs zero mean, we avoided the need for a bias term in our regressions. By making all features unit variance, we mitigate unequal treatment of features due to scaling differences when using ridge regression.

After fitting the observation model (encoding model) coefficients using ridge regression, we found the observation model noise covariance matrix R by dividing the sum of squared residuals matrix by degrees of freedom (number of data minus number of parameters).

4 FIT Kalman Filter

In our online comparisons, the Kalman filter used the position-as-feedback refinement of Gilja et al (2012) and the offline intention estimation refinement of Fan et al (2014).

The position-as-feedback refinement is implemented by: (1) setting the a priori estimate error covariance values (P'_t) for position to zero before the calculation of the Kalman gain; (2) setting the state transition model noise covariance (Q) entries for position to zero, except for the diagonal entries, which receive a small value (10^{-4}) for numerical stability. These two changes caused the Kalman gain to be calculated with the assumption that there is extremely small uncertainty in the position. This meant the filter attributed the innovation (actual spike counts minus predicted spike counts, $\bar{y}_t - H\tilde{s}_t'$) to errors in velocity only, and thus updated the velocity only. We did not use a precisely zero value for positional uncertainty to avoid singular matrix inversions. This refinement was only used for the FIT Kalman filter and only during closed-loop neural control of cursor.

Intention estimation is implemented as a pre-processing step before parameter fitting. The velocity vectors in the data for fitting the Kalman filter were rotated so that: (1) they pointed towards the target, when the cursor's center was outside the target; (2) they were zero, when the cursor's center was inside the target. This refinement was used in the FIT Kalman filter in closed-loop control as well as in encoding model analysis, where it was applied to both training and testing data for the Kalman Intention Estimation condition (only).

During closed-loop decoding, the position variable in the Kalman filter state integrates the previous time step velocity estimates via the transition model. To include the most recent velocity estimate in the position output, the most recent decoded velocity is added to the most recent decoded position estimate to get the final positional output for the FIT Kalman filter. The Kalman filter

parameters, both in offline reconstructions and closed-loop experiments, are fit on neural and hand movement data with zero temporal offset; like in Gilja et al. (2012), we did not optimize the temporal offset with strongest encoding.

5 Behavioral Tasks

We used two behavioral tasks. One task is the center-out cursor movement task (**Figure 2B**). Each trial consisted of the appearance of a circular target, reach toward the target, and hold duration. Successful holds were rewarded with a small amount of water. Each trial may have a peripheral or central target, and successive trials, if successfully completed, alternated central and peripheral targets, forming center-out-and-back movements. The center target was always at screen center (0, 0 position). Peripheral targets may appear at any location in an equal-distance ring around the screen center, with the exact location randomly chosen from a uniform distribution on angle. Circular targets were 5 cm in diameter (screen size) and the cursor was visually 2 cm in diameter, though it was treated as a point when deciding whether it was inside the target (i.e. logically 0 cm in diameter).

The distance from screen center to the center of peripheral targets was 10 cm during control of the cursor by hand movements and 8 cm during control of the cursor by neural activity. This was to increase the chances that hand movements in the training data covered cursor locations encountered during neural control, to make training data as representative as possible.

The hold time was fixed at 500 ms for all sessions. The inter-trial-interval was set to zero on successful trials. A 200 ms inter-trial-interval was used to penalize failed trials. Trials were considered a failure if the target was not entered 5000 ms after appearance. If the target was entered but hold time was insufficient, the failure timer was restarted from zero the moment the cursor exited the target. Thus it was possible for successful trials to be longer than 5000 ms.

Monkeys also performed a moving target pursuit task with the joystick, for offline analysis and reconstructions only. In this task, the 6 cm diameter target moved continuously following either a Lissajous curve (**Figure 2C**) or smoothed point-to-point trajectory (**Figure 2D**). The monkey had to keep the cursor within the moving target to receive periodic water rewards. The Lissajous curve was described by the following equations:

$$tx_t = A\sin(a \cdot v \cdot t + \delta), \tag{21}$$

$$ty_t = B\sin(b \cdot v \cdot t), \tag{22}$$

where tx_t and ty_t are the target x and y axis positions, respectively, in screen coordinates at time t, in seconds, and the Lissajous curve parameters were $a = 3$, $b = 4$, $v \in \{0.15, 0.2\}$ Hz, $\delta = 0.5\pi$, and $A = B = 7$cm. The temporal frequency was different for the x and y axes, making the values of the two coordinates uncorrelated. Our previous study showed that the UKF1's movement model alone could not reconstruct the Lissajous curve (Li et al. 2009), so even though the curve is deterministic, neural decoding was required to trace it with a cursor under UKF1 control.

For the point-to-point trajectory, the target moved smoothly between points uniformly drawn from the screen workspace. These waypoints were visible to the monkey, so that it could plan movements. The target's acceleration (≤ 3 cm/s^2, screen units) and velocity (≤ 6 cm/s, screen units)

were limited to create smooth target trajectories. This task has similarities with the instructed path task (Sadtler et al. 2015), but adds velocity requirements.

6 References

Fan, J. M., Nuyujukian, P., Kao, J. C., Chestek, C. A., Ryu, S. I., and Shenoy, K. V. (2014), Intention estimation in brain-machine interfaces, *Journal of Neural Engineering*, 11, 1, 016004, doi: 10.1088/1741-2560/11/1/016004

Gilja, V., Nuyujukian, P., Chestek, C. A., Cunningham, J. P., Yu, B. M., Fan, J. M., et al. (2012), A high-performance neural prosthesis enabled by control algorithm design, *Nat Neurosci*, 15, 12, 1752–1757, doi: 10.1038/nn.3265

Julier, S., Uhlmann, J., and Durrant-Whyte, H. F. (2000), A new method for the nonlinear transformation of means and covariances in filters and estimators, *IEEE Transactions on Automatic Control*, 45, 477–482

Julier, S. J. (2002), The scaled unscented transformation, in Proceedings of the American Control Conference, volume 6, volume 6, 4555–4559

Julier, S. J., Uhlmann, J. K., and Durrant-Whyte, H. F. (1995), A new approach for filtering nonlinear systems, in Proceedings of the American Control Conference, volume 3, volume 3, 1628–1632

Li, Z., O'Doherty, J. E., Hanson, T. L., Lebedev, M. A., Henriquez, C. S., and Nicolelis, M. A. L. (2009), Unscented Kalman filter for brain-machine interfaces, *PLoS ONE*, 4, 7, e6243, doi: 10.1371/journal.pone.0006243

Sadtler, P. T., Ryu, S. I., Tyler-Kabara, E. C., Yu, B. M., and Batista, A. P. (2015), Brain-computer interface control along instructed paths, *Journal of Neural Engineering*, 12, 1, 016015, doi: 10.1088/1741-2560/12/1/016015

Tarpey, T. (2000), A note on the prediction sum of squares statistic for restricted least squares, *The American Statistician*, 54, 2, 116–118

Welch, G. and Bishop, G. (2006), An introduction to the Kalman filter, Technical Report 95-041, University of North Carolina at Chapel Hill

7 Supplementary Table

(See next page.)

TABLE S1 | Decoder and preprocessing summary.

	Common to all three decoders	UKF2	Kalman	UKF1
State variables	2 dimensional task	Hand position, hand velocity, hand acceleration, target position, 5 taps (40 variables)	Hand position, hand velocity (4 variables)	Hand position, hand velocity, 10 taps (40 variables)
Observation model features	All features' coefficients are fitted using ridge regression with automatic optimization of ridge parameter using the PRESS statistic	Hand position, hand distance from screen center, hand velocity, velocity magnitude, acceleration, acceleration magnitude, target position, hand to target distance, position-velocity multiplicative terms: 5 taps, population history (70+n features)	Hand position, hand velocity (4 features)	Hand position, hand distance from screen center, hand velocity, velocity magnitude, 10 taps (60 features)
Offline reconstruction	Smoothing of training and testing position data (width 11 windowed moving average), movement models fitted to data	Did not use position-velocity mixing, velocity thresholding, or cursor control by future predictions	Did not use position-as-feedback or intention estimation	
Encoding model analysis	1 tap, bias term instead of centering of data	Did not use position-velocity mixing, velocity thresholding, or cursor control by future predictions	With and without intention estimation	
Closed-loop control	Movement models designed based on physical laws	Used position-velocity mixing for cursor control, velocity thresholding, cursor control by future predictions, smoothing of training data (width 5 sliding window moving average, applied to position and velocity)	Used position-as-feedback, intention estimation, making it the FIT Kalman filter	
